# Multiple mitochondria-targeted components screened from Sini decoction improved cardiac energetics and mitochondrial dysfunction to attenuate doxorubicin-induced cardiomyopathy

**DOI:** 10.7150/thno.80066

**Published:** 2023-01-01

**Authors:** Xin Ding, Ya Zhang, Pengchao Pan, Cuiping Long, Xingxing Zhang, Lingxin Zhuo, Qian Zhou, Wenting Liao, Guangguo Tan

**Affiliations:** 1 School of Pharmacy, Air Force Medical University, Xi'an 710032, China.; 2 School of Pharmacy, Shaanxi University of Chinese Medicine, Xi'an 712046, China.; 3 School of Pharmacy, Naval Medical University, Shanghai 200433, China.; 4 Department of Cardiovascular Medicine, the First Naval Hospital of Southern Theater Command, Zhanjiang 524005, China.; 5 School of Pharmacy, China Pharmaceutical University, Nanjing 210009, China.; 6 Department of traditional Chinese medicine, Xijing Hospital, Air Force Medical University, Xi'an 710032, China.

**Keywords:** Doxorubicin, Sini decoction, Cardiac mitochondria, Two-dimensional biochromatography, Multiomics

## Abstract

**Rationale**: Sini decoction (SND) is an efficient formula against DOX-induced cardiomyopathy (DCM), but the active ingredient combination (AIC) and mechanisms of SND remain unclear. Therefore, the present study aimed to identify the AIC and elucidate the underlying mechanism of AIC on DCM.

**Methods**: The AIC were screened by a novel comprehensive two-dimensional cardiac mitochondrial membrane chromatography (CMMC)-TOFMS analysis system and further validated by cell viability, reactive oxygen species (ROS) generation, ATP level, and mitochondrial membrane potential in DOX-induced H9c2 cell injury model. Then, an integrated model of cardiac mitochondrial metabolomics and proteomics were applied to clarify the underlying mechanism *in vitro*.

**Results**: The CMMC column lifespan was significantly improved to more than 10 days. Songorine (S), neoline, talatizamine, 8-gingerol (G) and isoliquiritigenin (I), exhibiting stronger retention on the first-dimension CMMC column, were screened to have protective effects against DOX cardiotoxicity in the H9c2 cell model. S, G and I were selected as an AIC from SND according to the bioactivity evaluation and the compatibility theory of SND. The combined *in vitro* use of S, G and I produced more profound therapeutic effects than any component used individually on increasing ATP levels and mitochondrial membrane potential and suppressing intracellular ROS production. Moreover, SGI attenuated DCM might via regulating mitochondrial energy metabolism and mitochondrial dysfunction.

**Conclusions**: The provided scientific evidence to support that SGI combination from SND could be used as a prebiotic agent for DCM. Importantly, the proposed two-dimensional CMMC-TOFMS analytical system provides a high-throughput screening strategy for mitochondria-targeted compounds from natural products, which could be applied to other subcellular organelle models for drug discovery.

## Introduction

Doxorubicin (DOX) is a widely used chemotherapy agent for treating solid tumours and leukaemia, but its clinical use is severely limited by dose-dependent cardiotoxicity 1. Dexrazoxane is the only clinically approved cardioprotectant against DOX cardiotoxicity 2. However, dexrazoxane-associated risk for secondary malignancies has been shown, which has resulted in its removal from the European market 3. Therefore, there is an urgent need to find alternative efficacious and safe strategies for DOX-induced cardiomyopathy (DCM).

Traditional Chinese medicines (TCM) have shown great potential for the discovery of drugs, which provides an opportunity to find new medications for DCM from TCM 4. Sini decoction (SND) is a famous TCM formula officially recorded in the Chinese pharmacopoeia for cardiovascular syndrome, which is composed of *Acontium carmichaelii, Zingiber officinale and Glycyrrhiza uralensis* with a mass ratio of *3:2:3* 5, 6. Studies from our group and others have indicated that SND effectively alleviates the symptoms of DCM and restores the perturbed metabolic homeostasis 5-8. Nevertheless, the cardioprotective components in SND and the mechanisms of alleviation of DCM still need further study.

Increasing evidence suggests that cardiac mitochondria are a prime target for DCM, and preventing mitochondrial dysfunction is an important therapeutic strategy for DCM 9. Mitochondria are intracellular double membrane-bound organelles that are ubiquitously found in eukaryotic cells and execute diverse functions, including adenosine triphosphate (ATP) generation, calcium homeostasis, and apoptosis regulation 10, 11. Mitochondrial dysfunction has been related to specific receptors expressed on the outer and inner mitochondrial membranes, and mitochondria have become a prominent intracellular pharmacological target for the development of new anti-DCM drugs 12, 13. Therefore, finding the active components from SND that interact with the mitochondrial membrane is promising for the treatment of DCM.

Biomembrane chromatography, which immobilizes biologically related membrane receptors on the solid support as a stationary phase, provides a powerful tool for screening bioactive compounds from complex matrices by chromatographic separation, in which the compound with longer retention times exhibits a stronger binding affinity to membrane receptors 14, 15. Cell membrane chromatography (CMC) is an effective biomembrane chromatographic technique, and our research group has established a suite of CMC models to screen active components in more than 40 kinds of TCM 16. It should be noted that the CMC models focus on components acting on cell membrane receptors and neglect the active components acting on intracellular organelles from TCM. In addition, the immobilization of biomembrane fragments on stationary phase carriers traditionally utilizes hydrogen bonds and electrostatic forces, which result in the fall-off of biomembrane fragments and further lead to a sharp decline in bioactivity and column life 17. Consequently, it is urgently needed to develop a stable mitochondrial membrane chromatography method to screen specific active components interacting with intracellular mitochondria from TCM. On the other hand, mitochondria are metabolic organelles and play a central role in regulating cellular respiration and energy metabolism 18, 19. The precise characterization of the mitochondrial proteome and metabolome without interference from other organelles would be helpful to better understand the pathogenesis of DCM and dissect the underlying mechanisms of the cardioprotective effects of mitochondria-targeted components from TCM.

Therefore, this work was initially designed to develop a new cardiac mitochondrial membrane chromatography (CMMC) method combined with two-dimensional liquid chromatography (2D-LC) / time-of-flight mass spectrometry (TOFMS) to screen active components targeting mitochondria to counteract DCM from SND, in which cardiac mitochondrial membrane fragments were immobilized onto 3-aminopropyltriethoxysilane (APTES)-modified silica by an amine-aldehyde condensation reaction to construct a new and stable mitochondrial membrane stationary phase (MMSP). The reproducibility and lifespan of the stationary phase were evaluated by using a ligand of the outer mitochondrial membrane translocator protein (TSPO). A pharmacological verification trial was further performed to validate their cardioprotective effects. Based on the results of pharmacological verification *in vitro* and the compatibility theory of SND, songorine (S), 8-gingerol (G) and isoliquiritigenin (I) were subsequently selected as representative active ingredients of *Acontium carmichaelii, Zingiber officinale* and *Glycyrrhiza uralensis*, respectively, and the efficacies and mechanisms of the SGI combination on DCM were tested in *in vitro* and* in vivo* experiments combined with an integrated model of cardiac mitochondrial metabolomics and proteomics.

## Materials and methods

All animal experimental procedures were carried out in accordance with the Administrative Committee on the Care and Use of Laboratory Animals at Air Force Medical University.

### Reagents

Details on *A. carmichaelii, Z. officinale* and *G. uralensis* and other materials used can be found in the Supplementary Materials and Methods.

### Preparation of SND and standard solutions

The dry herb pieces of *A. carmichaelii* 120 g, *Z. officinale* 80 g and *G. uralensis* 120 g (3:2:3) were mixed and extracted twice with boiling water (1:10 and then 1:8) for 2 and 1 h and then filtered by gauze. The extracting solution was combined and condensed to obtain SND with a concentration of 1.0 g/mL (expressed as raw materials). The LC-MS chemical profile of SND is provided in our previously published literature 20. A mixed test solution of 4'-chlorodiazepam (a positive drug targeting outer mitochondrial membrane translocator protein) and ranitidine (a negative drug) was prepared in methanol at a concentration of 1 mmol/L each.

### Isolation of cardiac mitochondria

Cardiac mitochondria were isolated using a modification of a published protocol 21. Briefly, male C57BL/6J mouse (weighing 22-26 g, Air Force Medical University, Xi'an, China) hearts were minced, incubated with trypsin and then homogenized with a Dounce glass homogenizer in isolation buffer (225 mM mannitol, 75 mM sucrose, 0.5% BSA, 0.5 mM EGTA and 30 mM Tris-HCl, pH 7.4). The homogenates were centrifuged at 800 x *g* at 4 °C for 10 min to remove nuclei and cellular debris. The supernatants were centrifuged at 9000 × *g* for 10 min, and the pellet containing the mitochondria was collected. The pellet was then washed and centrifuged at *9000 x g* at 4 °C for 10 min before resuspension for mitochondrial membrane preparation or proteomic and metabolomics analyses. Forty-five mouse hearts were collected and pooled every time to isolate cardiac mitochondria for mitochondrial membrane preparation.

### Preparation of cardiac mitochondrial membranes for CMMC

To obtain cardiac mitochondrial membranes, the freshly isolated mitochondria were further purified through Percoll density gradient centrifugation at 95000 × *g* for 30 min as previously reported 22. Pure mitochondria were washed twice by centrifugation at 6300×*g* for 10 min and resuspended in MRB (MRB: 250 mM mannitol, 5 mM HEPES and 0.5 mM EGTA, pH 7.4). The purity of isolated mitochondria was assessed by transmission electron microscopy (TEM) and western blotting. The experimental procedures are described in the Supplementary Materials and Methods. Mitochondria membranes were obtained from freshly prepared intact mitochondria by sonication five times for 2 s with 20 s intervals on ice with an ultrasonic cell disruptor. The suspension was centrifuged for 10 min at 12000 × *g* to remove unbroken mitochondria. The supernatant was centrifuged at 100000 × *g* for 60 min, and the pellet containing the mitochondrial membranes was collected. Then, 5 mL of saline was added to the pellet, and the cardiac mitochondrial membrane suspension was obtained. The mitochondrial membrane protein concentration was measured using a bicinchoninic acid (BCA) assay in duplicate.

### Synthesis and characterization of MMSP

**Figure 1:**
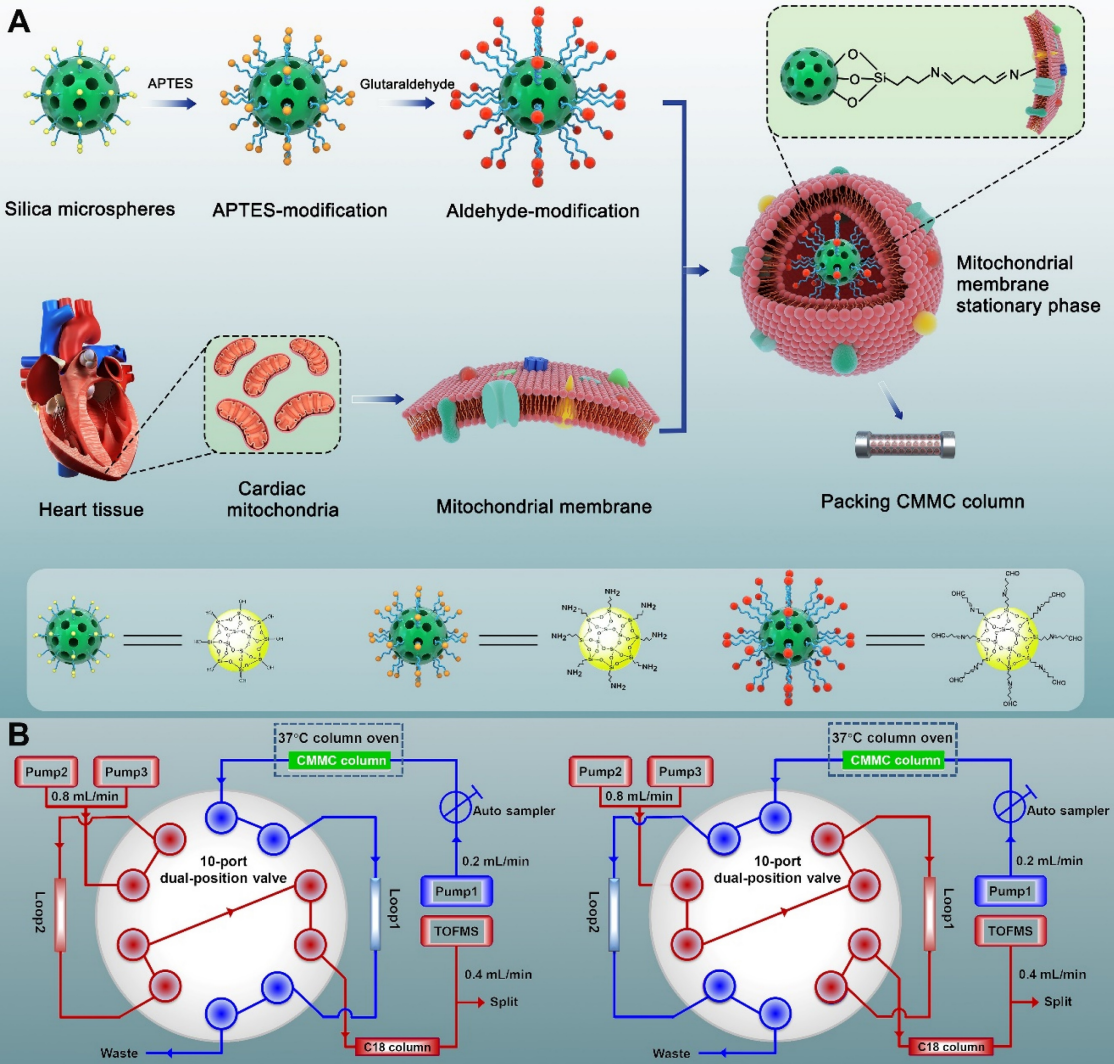
Synthesis process of covalent modified cardiac mitochondrial membrane stationary phase (**A**) and brief scheme of 2D CMMC/C18-TOFMS analytical system (**B**).

The synthesis process is shown in Figure 1A. Briefly, to obtain amino groups on the surface of silica gel,1 mL APTES and 2 g silica gel (5 μm, 200 Å) were mixed and stirred for 12 h in 100 mL toluene under nitrogen protection at 110 °C. Then, the suspension was washed three times with toluene by 5000 × *g* centrifugation. The sediment was subsequently reacted with 5% glutaraldehyde to obtain aldehyde-modified silica in 500 mL methanol solution at room temperature for 2 h. Finally, the aldehyde-modified silica (0.04 g) was reacted with cardiac mitochondria membranes through 5 min of vortexing in vacuum and 12 h of incubation at 4 °C to obtain MMSP, in which the free aldehyde groups on the other end of glutaraldehyde formed Schiff-base linkages with the amino groups on the surface of mitochondrial membranes via an amine-aldehyde condensation reaction. The morphology of MMSP was characterized by high-resolution field emission transmission electron microscopy (HRTEM, FEI Talos F200X TEM). The synthesized MMSP was packed by the wet packing method into a chromatographic column (10 × 2 mm, I.D.) according to our previous research 23.

### 2D CMMC analysis

A brief scheme of the 2D CMMC system is shown in Figure 1B. The system was conducted on an Agilent 1200 HPLC equipped with binary pumps (pumps 2 and 3) and a solvent degasser, autosampler, and column compartment controlled by an Agilent MassHunter Workstation (Agilent Technologies, Palo Alto, CA, USA). The in-house packed CMMC column was used as the first-dimension column, and the mobile phase was 10 mmol/L ammonia acetate at a flow rate of 0.2 mL/min (pump 1). A Capcellpak C18 column (100 mm × 3.0 mm, 3 µm, Shiseido, Japan) was applied for the second-dimension separation, and the mobile phase consisted of 0.1% formic acid (solvent A) and acetonitrile (solvent B) at 0.8 mL/min with a linear gradient elution program (10 to 60% B at 0 to 8 min; 60% B at 8 to 10 min; 60 to 10% B at 10 to 10.01 min;10% B at 10.01 to 13 min). A Rheodyne MXP9960-000 2-position/10-port switching valve (Rohnert Park, CA, USA) containing two 500 μL sample loops was used to connect the two dimensions. The output from the second-dimension column with a split ratio of 1:1 was introduced to the electrospray ionization (ESI) source of the Agilent 6220 Accurate-Mass Time-of-Flight LC/MS system (Agilent Technologies, Palo Alto, CA, USA). The detailed operation of the 2D system and MS conditions are described in our group's previous research 20, 24.

### Cell culture and viability assay

H9c2 rat cardiomyocyte cell lines were obtained from the cell bank of the Chinese Academy of Sciences (Shanghai, China). Cells were maintained with Dulbecco's modified Eagle's medium (DMEM) containing 10% foetal bovine serum (Gibco, New Zealand) supplemented with 1% penicillin-streptomycin. H9c2 cells were cultured in a 5% CO_2_ incubator at 37 °C. H9c2 rat cardiomyocyte cells, seeded at densities of 4.5 × 10^4^ cells/mL into 96-well plates, were pretreated with different concentrations of six weakly retained components and five strongly retained components for 6 h, followed by 2 μM DOX for 18 h to generate the cell injury model. Cell viability was then tested by CCK-8 assay (Beyotime Biotechnology). 10 µL of CCK-8 solution was added to each well and incubated at 37 °C for 2 h. The resulting colour was determined at 450 nm using an AMR-100 microplate reader (Hangzhou Allsheng Instruments Co., Ltd., China). All data are expressed as the means ± standard deviations (SD). The significant differences were assessed by one-way analysis of variance (ANOVA) followed by Dunnett's multiple comparison tests.

### *In vitro* experiments on the SGI combination

According to the cell viability assay and the compatibility theory of SND, S, G and I were selected as a representative active ingredients of *A. carmichaelii*, *Z. officinale* and *G. uralensis*, respectively, and the efficacy of SGI on DOX-induced H9c2 cell injury and antineoplastic activity of DOX towards two kinds of human carcinoma cell lines (HepG2 and K562 cells) were further evaluated, in which the composition mass ratio of SGI of 3:2:3 was selected as the optimal mass ratio of *A. carmichaelii-Z. officinale*-*G. uralensis*, and different concentrations of SGI were applied for 6 h before DOX treatment in all experiments. The viability was determined by the CCK-8 assay following the above procedures. The detailed experimental procedures to determine the antineoplastic activity of DOX towards HepG2 and K562 cells under the cotreatment of SGI can be found in the Supplementary Materials and Methods.

### Intracellular ROS detection

To detect intracellular reactive oxygen species (ROS), the cells were preloaded with 10 μM 2',7'-dichlorofluorescin diacetate (DCFH-DA, Beyotime Biotechnology, Shanghai, China) for 20 min at 37 °C. Afterwards, the dye was removed, the cells were rinsed twice with PBS, and a fluorescence microscope was used for fluorescence image collection. ImageJ software was used for quantification.

### ATP assays

Intracellular ATP levels were determined using an Enhanced ATP Assay Kit (Beyotime Biotechnology, Shanghai, China). The lysed cells were centrifuged for 5 min at 4 °C and 12,000 × *g*, and the supernatant was collected. Before ATP detection, detecting solution was added to a 96-well plate and incubated at room temperature for 5 min. The supernatant was then added to the plate, mixed quickly, and read within 30 min. The concentration of ATP was calculated according to an ATP calibration curve.

### Mitochondrial membrane potential (ΔΨm)

JC-1 dye was used to measure ∆Ψm in DOX-exposed and SGI-treated H9c2 cells. The detailed experimental procedures can be found in the Supplementary Materials and Methods.

### *In vivo* experiments on the SGI combination Animal studies

Fifty-five adult male C57BL/6J mice (weighting 22-26 g) were provided by the Laboratory Animal Center of Air Force Medical University (Xi'an, China) and housed in a standard environment (temperature 25 ± 2 °C, light/dark cycle, 12 h/12 h, humidity 55 ± 10%). The mice were randomly divided into three groups: control (0.3% CMC-Na, n=15), DOX (0.3% CMC-Na, n=20), and SGI-treated (n=20) groups. DOX was administered as an intraperitoneal injection of 6 mg/kg weekly for 4 weeks. SGI-treated animals received both DOX injections and SGI feed at a daily dose of 8 mg/kg body weight with a mass ratio of S-G-I of 3:2:3 over a period of 4 weeks. Weight was measured once weekly to monitor weight loss. Cardiac function was assessed using a Vevo 2100 echocardiography system Imaging System (VisualSonics, Toronto, Canada) as previously reported 25 at 4 weeks after the start of treatment. Blood samples were then collected for biomedical measurement, including myocardial marker enzymes creatine kinase (CK), creatine kinase MB (CK-MB) and lactate dehydrogenase (LDH), and the hearts were rapidly excised. Under random sampling, ten hearts in every experimental group were immediately used to extract cardiac mitochondria to perform metabolomic and proteomics assays, and the other hearts in every group were fixed with 10% buffered formalin and 2.5% glutaraldehyde for histopathology examination and TEM assay, respectively.

### Histological assessment of myocardial damage

Paraffin-embedded hearts were cut into 4- to 5-μm-thick tissue sections and then stained with haematoxylin/eosin (H&E) to assess the extent of vacuolation, interstitial oedema and loss of nuclei. Fibrosis was detected with Masson's trichrome staining (Sigma-Aldrich) according to the manufacturer's protocol. The collagen volume fraction (ratio of blue dye area to red dye area, CVF%) was calculated using ImageJ software.

### TEM assay

Heart ultrastructural analysis was performed to analyse and quantify mitochondrial structural changes. Sample preparation and conventional electron microscopy were performed as described previously 26. Briefly, the samples were fixed with 2.5% glutaraldehyde, fixed with 1% osmium tetroxide, subsequently dehydrated by a gradient series of acetone and embedded in Epon resin. Finally, ultrathin sections (70 nm) were cut and stained with uranyl acetate followed by lead citrate. Electron microscopy micrographs of thin sections were captured with a JEOL JEM-1230 TEM to evaluate the distribution and morphology of mitochondria. The cristae density of individual mitochondria was measured using ImageJ software.

### Sample preparation for mitochondrial proteomic and metabolomic analysis

Isolated mitochondria from each heart were resuspended in 150 μL MRB. The mitochondrial suspension was split into two aliquots for metabolomic and proteomic analysis.

For metabolomic analysis, the suspension was further split for protein quantification by BCA assay (20 μL) and metabolomic analysis (50 μL). Fifty microlitres of mitochondrial resuspension was centrifuged at 11000 × *g* at 4 °C for 10 min to remove the MRB solution, and the pellet was resuspended in 100 μL acetonitrile by vortexing (1 min) and then sonicated for 10 min in an ice bath, followed by 12000 × *g* centrifugation for 15 min at 4 °C. The clear supernatants were transferred to LC autosampler vials for ultra-high-performance liquid chromatography (UHPLC)-high resolution MS-based metabolomic analysis. A pooled sample prepared by mixing aliquots of individual samples was used as a quality control (QC) sample to monitor the performance of metabolomics workflows in our lab 27.

For proteomic analysis, 75 μL of mitochondrial resuspension was centrifuged at 11000× *g* at 4 °C for 10 min to remove MRB solution, the pellet was resuspended in 50 μL RIPA lysis buffer by vortexing (1 min) and then sonicated for 20 min in an ice bath, followed by 12000× *g* centrifugation for 15 min at 4 °C. The supernatants were collected, and the protein concentration was determined by BCA assay. Subsequently, equal aliquots from individual samples in the same group were mixed, and the pooled sample from each group was subjected to proteomic analysis in triplicate. Ten micrograms of protein from each pooled sample were subjected to 10% SDS‒PAGE separation, and then the gels were fixed and stained with colloidal Coomassie Blue. Three gel slices were excised from each lane, and the gel slices were then destained with 25% acetonitrile (ACN) in 50 mM ammonium bicarbonate. The proteins were reduced with 20 mM DTT and alkylated with 50 mM iodoacetamide. The proteins were digested by trypsin at a protein to enzyme ratio of 50:1 overnight at 37 °C. The resulting peptides were desalted with a Millipore C18 ZipTip column (Merck Millipore, Darmstadt. Germany) and then analysed by LC‒MS/MS-based proteomics.

### Metabolomics analysis

Metabolomic profiles of mitochondria were obtained with an ExionLC™ AD system coupled to an AB SCIEX X500R QTOF mass spectrometer (AB SCIEX, USA) operated in full-scan MS resolution mode in two separate acquisition-positive and negative ionization modes over a mass range of 100 to 1000 Da. Mitochondria metabolites were separated on an Agilent Infinity LabProroshell 120 PFP column (100 × 2.1 mm, 1.9 μm). More details of the LC‒MS conditions are provided in the Supplementary Materials and Methods.

The acquired raw LC-MS data (.wiff2) were converted to open-format “mzXML” files using the “msconvert” utility of the ProteoWizard toolkit (version 3.0.21188) 28. The files were then processed by the XCMS package in R (version 3.14.1) 29. The XCMS processing parameters were optimized and set as follows: mass accuracy for peak detection = 25 ppm; peak width c = (5, 30); snthresh = 5; bw = 10; mzwid = 0.015; minfrac = 0.5. The CAMERA package was used for subsequent peak annotation 30. The resulting data were cleaned up by MS-FLO to eliminate potential duplicates and isotopes 31. Features for which the coefficient of variation measured on repeated injections of the QC samples were larger than 30% or those missing values from at least 75% of measurements were excluded from the data matrix. The data matrix was divided by the total spectral intensity and then divided by the total protein content for normalization.

Multivariate and univariate statistical analyses were carried out using SIMCA-P software V14.1 (Umetrics, Umea, Sweden) employing principal component analysis (PCA) and orthogonal partial least squares-discriminant analysis (OPLS-DA) algorithms, in which the data were mean-centred and scaled by the Pareto method, and by SPSS software using one-way ANOVA followed by Dunnett's multiple comparison tests. Target *m/z* features with VIP (variable importance in the projection) value > 1 in the OPLS-DA model, a *p* value calculated from one-way ANOVA <0.05, FDR (false discovery rate) < 0.05 in multiple test corrections using the Benjamini-Hochberg procedure, and the fold change (ratio of abundance observed between control and DOX groups) >1.5 were adopted to evaluate statistical significance.

After statistical analysis, target *m/z* features were putatively annotated using the online METLIN and HMDB databases with a 10-ppm tolerance. Further structural elucidation was performed by comparison with the authentic standards available in our lab or MS/MS experiments. The MS/MS spectra were matched against spectral libraries from METLIN and HMDB that were compiled with either authentic standards or theoretical assignment. Metabolites for which MS/MS reference spectra were not available were annotated using chemical fragmentation rules.

In addition, the Kyoto Encyclopedia of Genes and Genomes (KEGG) database was applied to construct a metabolomic pathway network diagram. KEGG-based pathway enrichment analysis was performed to screen metabolomic pathways of the SGI-reversed metabolites using MetaboAnalyst 5.0 32, in which the metabolites without KEGG IDs were replaced with the annotated KEGG IDs that were structurally homologous.

### Label-free proteomics

Label-free proteomics was performed on a U3000 Ultimate nano LC system coupled with a Q-ExactivePlus MS instrument (Thermo Scientific). The peptides were separated on a C18 reversed-phase nanocolumn (AcclaimPepMap TM 100, 75 μm × 15 cm, 3 μm, 100 Å, Thermo Scientific) with a 70 min gradient at 0.35 μL/min. Details of the LC‒MS conditions for proteomics analysis are provided in the Supplementary Materials and Methods.

The LC-MS/MS data were analysed using a pipeline implemented in Proteome Discoverer software (Version. 2.3.0.523, Thermo Fisher Scientific, San Jose, USA) with the SEQUEST algorithm, in which the MS/MS data were searched against a SwissProt Mus musculus database (Swiss-Prot, downloaded on Mar 16, 2020). The following search parameters were used: the mass tolerances of precursor and product ions were 10 ppm and 0.02 Da, respectively; trypsin was defined as the protease, and up to two missed cleavages were allowed. Protein confidence indicators were thresholded at ≤ 1% (strict) and ≤ 5% (relaxed) FDR. Carbamidomethylation of cysteine residues was set as a fixed modification. Oxidation of methionine residues, acetylation of the protein N-terminus, and deamidation of asparagine and glutamine were treated as variable modifications. Label-free quantification of proteins required at least two ratio counts of unique peptides. Only unique peptides were used for quantification. To ensure accurate quantification, proteins identified in each sample were normalized to the summed intensity, and the data were log2-transformed. Differentially expressed proteins (DEPs) between different groups were screened out according to the following criteria: *p* value <0.05 (Student's two-tailed t test) and fold change >1.2 or <0.83. A volcano plot was generated for the DEPs based on the *p* value and fold change. One-way ANOVA was performed successively to reveal the significant differences in the variables among different groups.

The molecular mechanisms and functional activity of the DEPs were further elucidated according to Gene Ontology (GO) annotation, KEGG pathway and Wikipathway enrichment analysis by the String database 33.

### Integration of metabolomics and proteomics

To analyse the significantly SGI-regulated metabolic pathways, the SGI-reversed metabolites and proteins with significant differences between the DOX- and SGI-treated groups were subjected to joint pathway analysis using MetaboAnalyst 5.0. Fisher's exact test was used to perform enrichment analysis. In addition, integrated molecular pathway level analysis (IMPaLA) was performed for pathway overrepresentation analysis to obtain more information from other databases, including Wikipathways, Reactome, and KEGG 34.

### Western blot validation of differentially expressed protein

Western blot analysis was used to detect and quantify the protein expression of succinate dehydrogenase (Sdha), long-chain-fatty-acid-CoA ligase 1 (Acsl1),2-oxoglutarate dehydrogenase (Ogdh), carnitine O-palmitoyltransferase 1 (Cpt1b) and carnitine O-palmitoyltransferase 2 (Cpt2) in cardiac mitochondrial proteins. We also quantified the main mitochondrial dynamics-related proteins mitofusin 1 (Mfn1), mitofusin 2 (Mfn2) and isoform 2 of dynamin-like 120 kDa protein (Opa1). Cardiac mitochondrial total protein was extracted from the isolated cardiac mitochondria using RIPA buffer and quantified using the BCA Assay. Total protein was separated by SDS‒PAGE and transferred onto PVDF membranes. The membranes were blocked with 5% free-fat milk and subsequently incubated overnight at 4 °C with the respective primary antibodies. Subsequently, the membranes were incubated with secondary antibodies for 1 h. COX IV was the internal control. Finally, the target protein bands were visualized using an ECL chemiluminescence detection kit (Immobilon™ Western, Millipore Corporation, Billerica, USA) and analysed using Image Lab 5.0 software (Bio-Rad). The experiments were repeated in triplicate, and the quantification was normalized to the corresponding value of COX IV expression.

### Statistical analysis

Data are shown as the mean ± SD and were analysed using SPSS 20.0 software (IBM). One-way ANOVA followed by Dunnett's multiple comparison test was used to compare the groups. *p*< 0.05 was considered statistically significant.

## Results

### Purity of the isolated cardiac mitochondria

**Figure 2:**
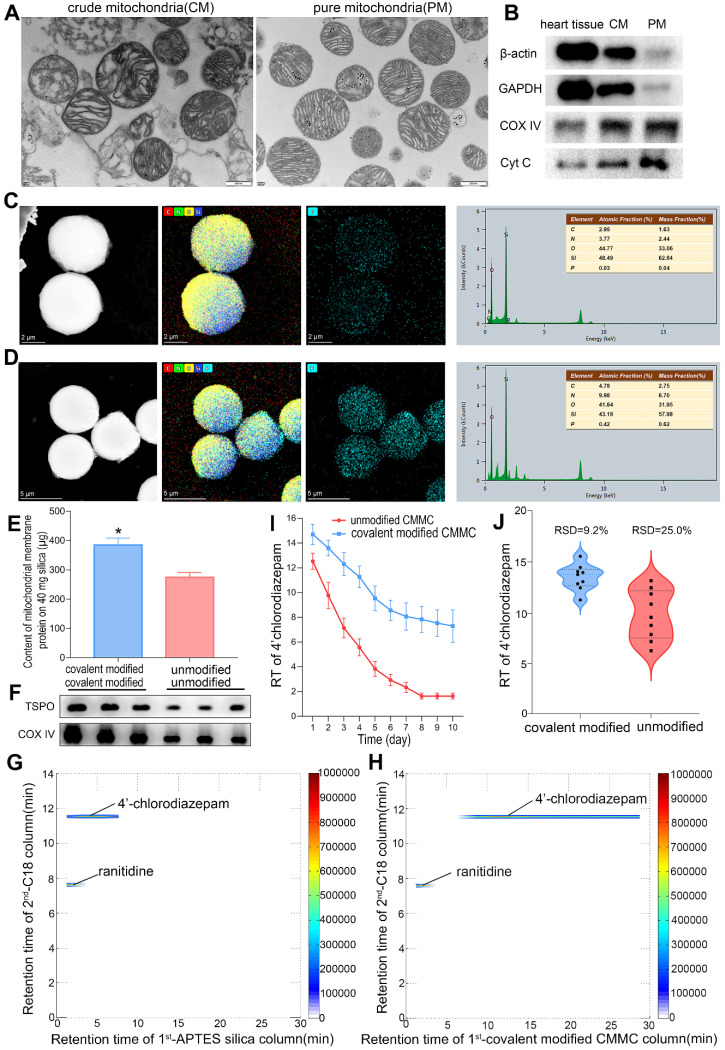
The purity assessment of purified mitochondria, and HRTEM image, protein content and blotting, selectivity, lifespan, and reproducibility of mitochondria membrane-coated APTES-silica stationary phase. (**A**) TEM showing typical mitochondrial morphology (20,500×). (**B**) Western blot analyses of subcellular marker proteins. (**C** and **D**) HRTEM image, corresponding EDX elemental mapping of phosphorus and energy spectrum of APTES-modified silica microparticles (**C**) and mitochondria membrane-coated APTES-silica microparticles (**D**). (**E**) Content of mitochondria membrane protein (μg) on 40 mg APTES-modified and unmodified silica. Data are expressed as means ± SD, n=3; *p<0.05, vs. unmodified group. (**F**) Western blotting of TSPO and COX IV proteins in mitochondria membrane-coated stationary phase. (**G** and **H**) Typical 2D contour plots of 4'-chlorodiazepam and ranitidine obtained by APTES silica column TOFMS system (**G**) and covalent modified CMMC column TOFMS system (**H**). (**I**) Retention time (RT) of 4'-chlorodiazepam on covalent modified and unmodified CMMC columns (n = 3). (**J**) The reproducibility (RSD) of RT of 4'-chlorodiazepam from unmodified and covalent modified CMMC column for the first 3 days.

The purity of the isolated cardiac mitochondria is essential for the successful development of MMSP. Representative transmission electron micrographs (TEM) of the isolated crude and pure mitochondria showed that pure mitochondria contained substantially fewer contaminants than crude mitochondria (Figure 2A), suggesting a high level of purity. The micrographs also showed that the inner and outer mitochondrial membranes and mitochondrial cristae were intact, confirming the high structural integrity of our isolated mitochondria. Western blotting of several subcellular markers was further performed to evaluate the purity, including mitochondrial proteins (cytochrome c (cyt c) and COX-IV) and cytosolic proteins (β-actin and GAPDH). As shown in Figure 2B, cytosolic proteins were effectively removed after Percoll density gradient centrifugation. Mitochondrial marker proteins were readily detected, indicating that whole mitochondria were greatly enriched. Structurally intact and high-purity mitochondria were obtained to develop MMSP. Considering that the low yield of purification would be unfavourable for the demands of mitochondrial metabolomics and proteomics, intact crude mitochondria were used for metabolomic and proteomic analysis.

### Selectivity, lifespan and reproducibility of the CMMC column

The morphological characteristics of MMSP were investigated by high-resolution field emission transmission electron microscopy (HRTEM). Figure 2C-D shows the HRTEM image, corresponding to EDX elemental mapping of phosphorus and the energy spectrum of APTES-silica and mitochondrial membrane-coated APTES-silica microparticles, confirming the enriched existence of phosphorus (P) originating from mitochondrial membrane proteins in MMSP. In addition, the BCA assay demonstrated that the contents of immobilized mitochondrial membrane protein on nonmodified and APTES-modified silica (40 mg) were 274.08 ± 17.47 μg and 384.37 ± 24.08 μg (Figure 2E), respectively. Moreover, after membrane coating procedures, a higher membrane protein immunoblot intensity of TSPO and COX IV was observed by western blot in APTES-modified silica than in nonmodified silica (Figure 2F). Therefore, we believed that mitochondrial membrane fragments were successfully anchored to APTES-silica based on covalent binding. The selectivity, reproducibility and lifespan of the CMMC column were evaluated using a positive drug (4'-chlorodiazepam, a ligand of the outer mitochondrial membrane translocator protein) and a negative drug (ranitidine). As shown in Figure 2H, 4'-chlorodiazepam showed strong retention behaviour on the CMMC column, while ranitidine was hardly retained. Moreover, 4'-chlorodiazepam and ranitidine had no retention on the APTES-silica column (Figure 2G). To evaluate the lifespan of the column, the retention time (RT) of 4'-chlorodiazepam on modified and unmodified CMMC columns was compared by three injections per day over 10 days. As shown in Figure 2I, the RT of 4'-chlorodiazepam on unmodified CMMC columns decreased sharply in the first 4 days and was near zero on the 7^th^ day owing to the loss of mitochondrial membrane fragments from the stationary phase. However, the RT on APTES-modified CMMC columns remained over 7 min on the 10^th^ day, suggesting that the binding stability of membrane fragments on APTES-silica was significantly increased compared with unmodified silica. The results indicated that the lifespan of the APTES-modified CMMC column was prolonged to at least 10 days. In addition, the reproducibility (relative standard deviation, RSD) also increased from 25.0% to 9.2% for the first 3 days when the RT fell quickly (Figure 2J). These results indicated that our developed CMMC system provided satisfactory selectivity, reproducibility and column lifespan for real sample analysis.

### Application of the 2D CMMC system to SND

**Figure 3:**
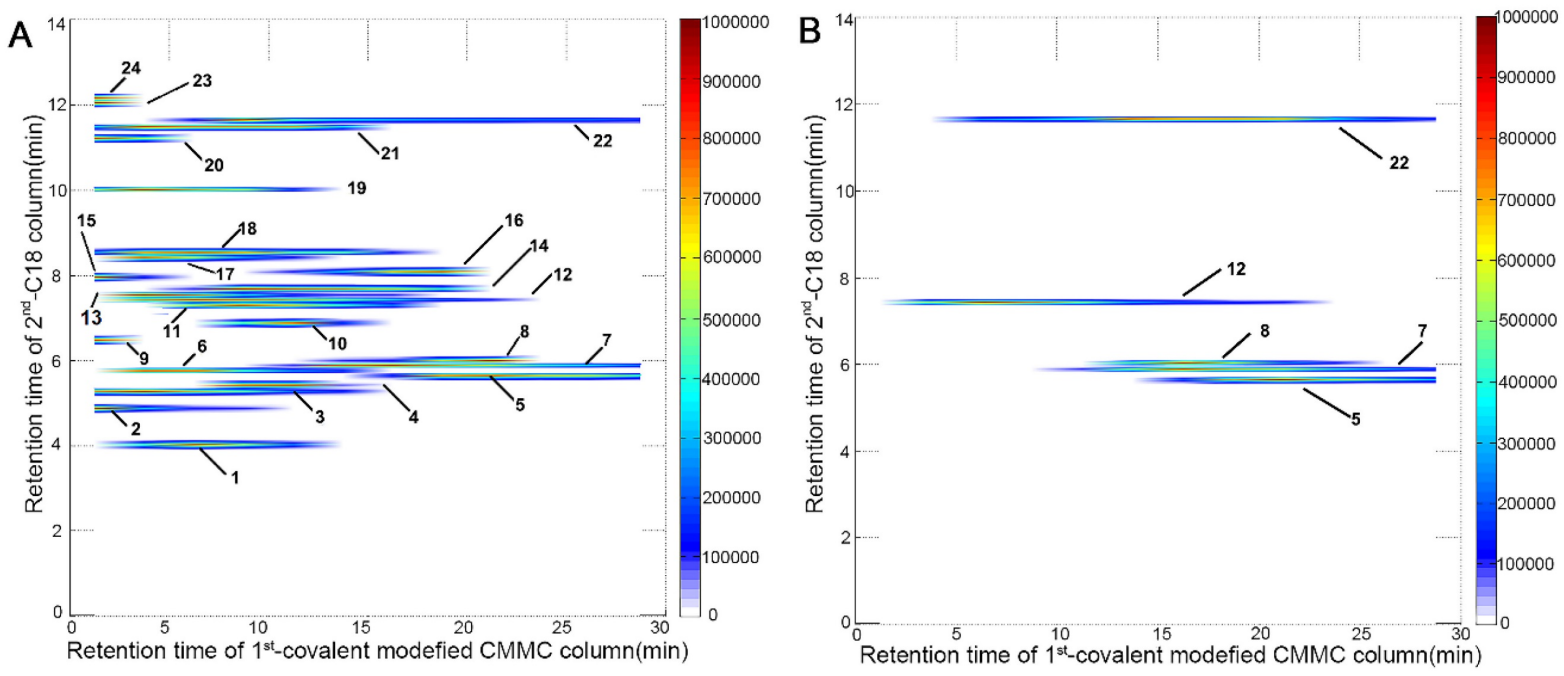
2D contour plots of SND (**A**) and five mixed standard solution including S (5), neoline (7), talatizamine (8), I (12) and G (22) (**B**) obtained by the 2D covalent modified CMMC/C18 column-TOFMS system.

The comprehensive 2D CMMC system was further employed to screen active components targeting mitochondria from SND to counter DCM. As shown in Figure 3A, 24 components were directly detected by the 2D contour spectrum obtained from the 2D analytical system and were credibly identified by C18 column-TOFMS according to in-house library matching and our previously reported TOFMS data 20. The identification results are shown in Supporting Information Table S1. Five components with strong retention on CMMC were identified as S (5), neoline (7), talatizamine (8), I (12), and G (22) (Table 1) and validated by their mixed standard solution. As shown in Figure 3B, the retention behaviours of these five standard compounds were similar to those in the SND analysis, indicating that these components could tightly bind with cardiac mitochondrial membranes, while their protective activities against DCM need further verification.

### Effect of potential active compounds on H9c2 cell viability

**Figure 4:**
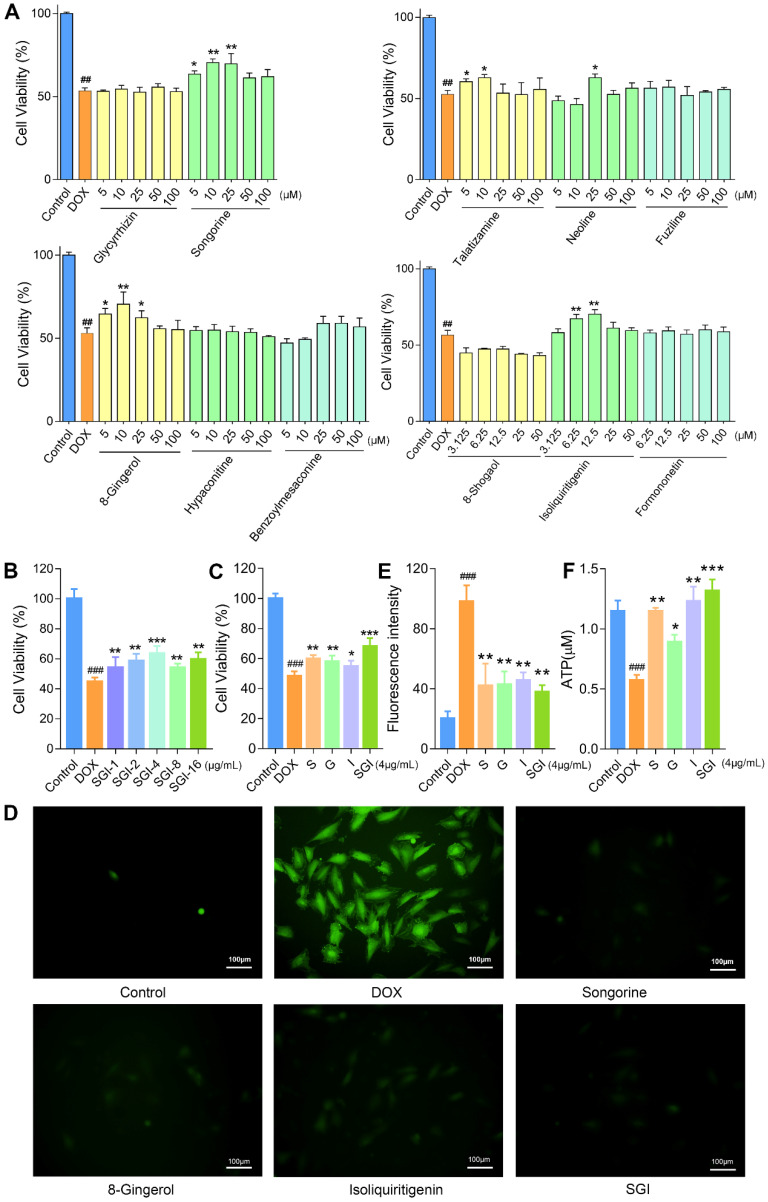
Effect of six weakly retained components, five strongly retained components and SGI combination on DOX-induced cell death, ROS generation and ATP level in H9c2 cells. (**A**) Cell viability (%) on exposure with various concentrations of six weakly retained and five strongly retained components in DOX-induced cell death model. (**B** and **C**) Cell viability (%) on exposure with various concentrations of SGI combination (**B**) and the comparison results between respective component and SGI combination (**C**). (**D**) ROS fluorescence images of SGI combination and respective component-treated groups (scale bar = 100 μm, 100 ×). (**E**) The ratio of ROS fluorescence intensity. (**F**) Intracellular ATP levels were regulated by SGI combination and respective component. ^##^*p* < 0.01, ^###^*p* < 0.001, compared with the control group; * *p* < 0.05, **p < 0.01, *** *p* <0.001, compared with the DOX group. Data are mean ± SD, n=5.

To validate the accuracy and validity of this 2D CMMC system, the DOX-induced H9c2 cell injury model was further applied to evaluate the bioactivities of these binding components, including six weakly retained components and five strongly retained components, with the first-dimension CMMC column (Figure 4A). The results indicated that the cell viability was decreased to approximately 53% with 2 μM DOX when compared to control cells. After pretreatment with six weakly retained components (fuziline, benzoylmesaconine, hypaconitine, glycyrrhizin at 5, 10, 25, 50, 100 μM and formononetin and 8-shogaol at 3.125, 6.25, 12.5, 25, 50 μM) and five strongly retained components (S, neoline, talatizamine, G at 5, 10, 25, 50, 100 μM and I (3.125, 6.25, 12.5, 25, 50 μM), the viability of cells in the S (5, 10 and 25 μM), neoline (25 μM), talatizamine (5, 10 μM), G (5, 10, 25 μM) and I (6.25,12.5 μM) treatment groups were significantly increased compared with the DOX group (*p* < 0.05). However, the six weakly retained components indicated little protective effects at the given concentrations (*p* > 0.05, compared to the DOX group). These* in vitro* results confirmed the cardioprotective effects of the five strongly retained components and the selectivity of the 2D CMMC system in screening potential components against DCM.

The combined results of the cell viability and retention behaviours on the CMMC column demonstrated that S, G, and I are the major active ingredients of *A. carmichaelii*, *Z. officinale* and *G. uralensis*, respectively. Based on the theories of TCM, the SND formula is designed to contain a combination of three kinds of plants to improve clinical efficacy. In the past few years, the pharmaceutical industry has witnessed a shift from the pursuit of “magic bullets” that hit a single target to seeking combination treatment that consists of multiple active ingredients 35. Therefore, in this study, we further focused on the efficacies and mechanisms of the SGI combination on DCM *in vitro* and* in vivo*.

### Effects of the SGI combination on cell viability, ROS, ATP, and ΔΨm *in vitro*

When cotreated with the SGI combination, the cell viability results showed that SGI provided a significant protective effect against DOX-induced cell death in a low dose-dependent manner (Figure 4B). The results at the optimal concentration of 4 μg/mL, containing 1.5 μg/mL S, 1 μg/mL G and 1.5 μg/mL I, also demonstrated that the SGI combination was clearly superior to each compound alone in increasing cell viability in H9c2 cells (Figure 4C).

A body of evidence suggests that excess production of ROS is the primary mechanism involved in DOX-induced cardiotoxicity 36. As shown in Figure 4D-E, intracellular ROS production was significantly increased in the DOX-treated H9c2 cells compared with the control group, whereas pretreatment with S, G, and I, alone or combined, significantly decreased the generation of ROS. Moreover, the SGI combination was significantly superior to each compound alone in inhibiting DOX-induced intracellular ROS generation.

**Figure 5:**
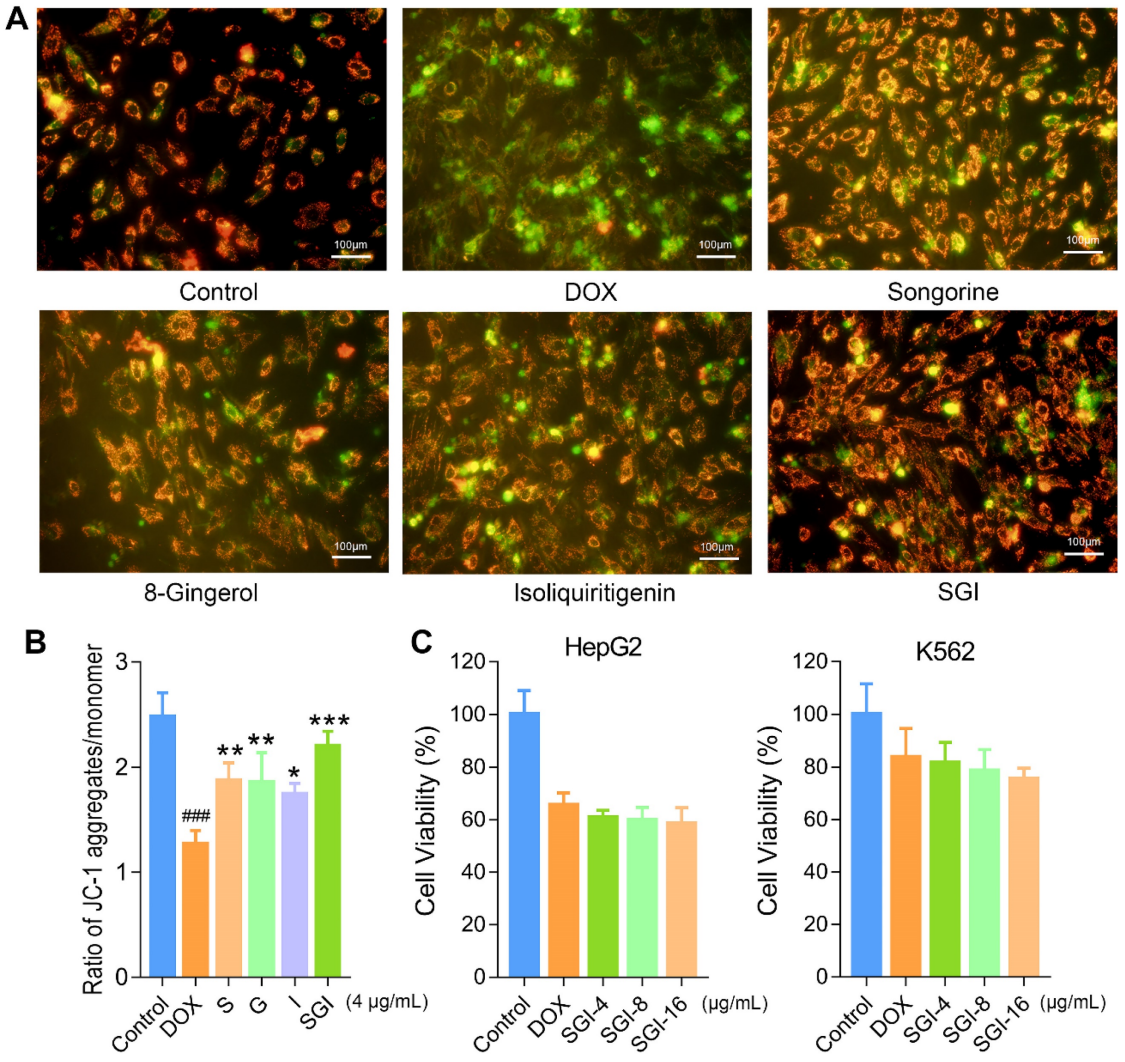
Effect of and SGI combination and respective component on dissipation of ΔΨm in H9c2 cells and effect of SGI combination on DOX's antineoplastic activity. (**A**) The fluorescence images of SGI combination and respective component-treated groups (scale bar = 100 μm, 100 ×). Red fluorescence represents the mitochondrial aggregate form of JC-1, indicating intact mitochondrial membrane potential. Green fluorescence represents the monomeric form of JC-1, indicating dissipation of ΔΨm. (**B**) Ratio of red fluorescence to green fluorescence. (**C**) Effect of SGI combination on DOX's antineoplastic activity. HepG2 and K562 cell viabilities after DOX treatment for 24 h with or without pre-treatment of different concentrations of SGI. ^###^ *p* < 0.001, compared with the control group; * *p* < 0.05, ** *p* < 0.01, *** *p* < 0.001, compared with the DOX group. Data are mean ± SD, n = 5.

Subsequently, mitochondrial function was evaluated by determining the ATP level and ΔΨm. Our data suggested that DOX treatment caused a decrease in intracellular ATP synthesis, and pretreatment with S, G, and I could significantly increase the DOX-induced reduction in intracellular ATP content (Figure 4F). Likewise, these results suggested that the SGI combination intensified the therapeutic efficacy. We further used the JC-1 method to measure the ΔΨm in cells, as JC-1 dye could accumulate in normal mitochondria and present red fluorescence. Exposure of H9c2 cells to DOX (1 μM) for 18 h led to dissipation of ΔΨm, indicating increased green fluorescence after JC-1 staining. Pretreatment with SGI (4 μg/mL) neutralized the dissipation, showing the protective effect of SGI (Figure 5A). The ratio of red and green fluorescence was further employed to assess the toxicity of DOX to mitochondria and the protective effect of SGI (Figure 5B). In normal cells, JC-1 accumulated in mitochondria with a ratio of 2.48 ± 0.23. Cells exposed to DOX showed a lower ratio (1.27 ± 0.13, *p* < 0.05, compared to the control group, n = 6) because JC-1 was distributed throughout the cytosol in its monomeric form, which demonstrated the dissipation of ΔΨm. Cells pretreated with the SGI combination showed attenuation of the dissipation of ΔΨm (2.20 ± 0.14, *p* < 0.05, compared to the control and DOX groups, n = 6). Similar to the cell viability assay, the SGI combination was significantly superior to each compound alone in increasing ΔΨm (Figure 5A-B). Similarly, these results suggested that the SGI combination intensified the therapeutic efficacy.

### Effect of the SGI combination on the antineoplastic activity of DOX *in vitro*

To verify that the SGI combination did not interfere with the antineoplastic activity of DOX, HepG2 and K562 cells were employed. The viabilities of HepG2 and K562 cells pretreated with the SGI combination were 60.82 ± 2.81% and 81.62 ± 7.78%, 59.78 ± 4.94% and 78.54 ± 8.12%, 58.57 ± 6.05% and 75.40 ± 4.24% for the 4 μg/mL, 8 μg/mL and 16 μg/mL SGI combinations, respectively. As shown in Figure 5C, no significant difference could be observed when compared to DOX treatment alone (65.57 ± 4.61% and 83.81 ± 10.74%). It was found that DOX seemed to exhibit a stronger antineoplastic effect when treated with a high concentration of the SGI combination. These results suggested that the SGI combination did not interfere with the antineoplastic activity of DOX on these two cancer cell lines.

### SGI combination attenuated DCM *in vivo*

**Figure 6:**
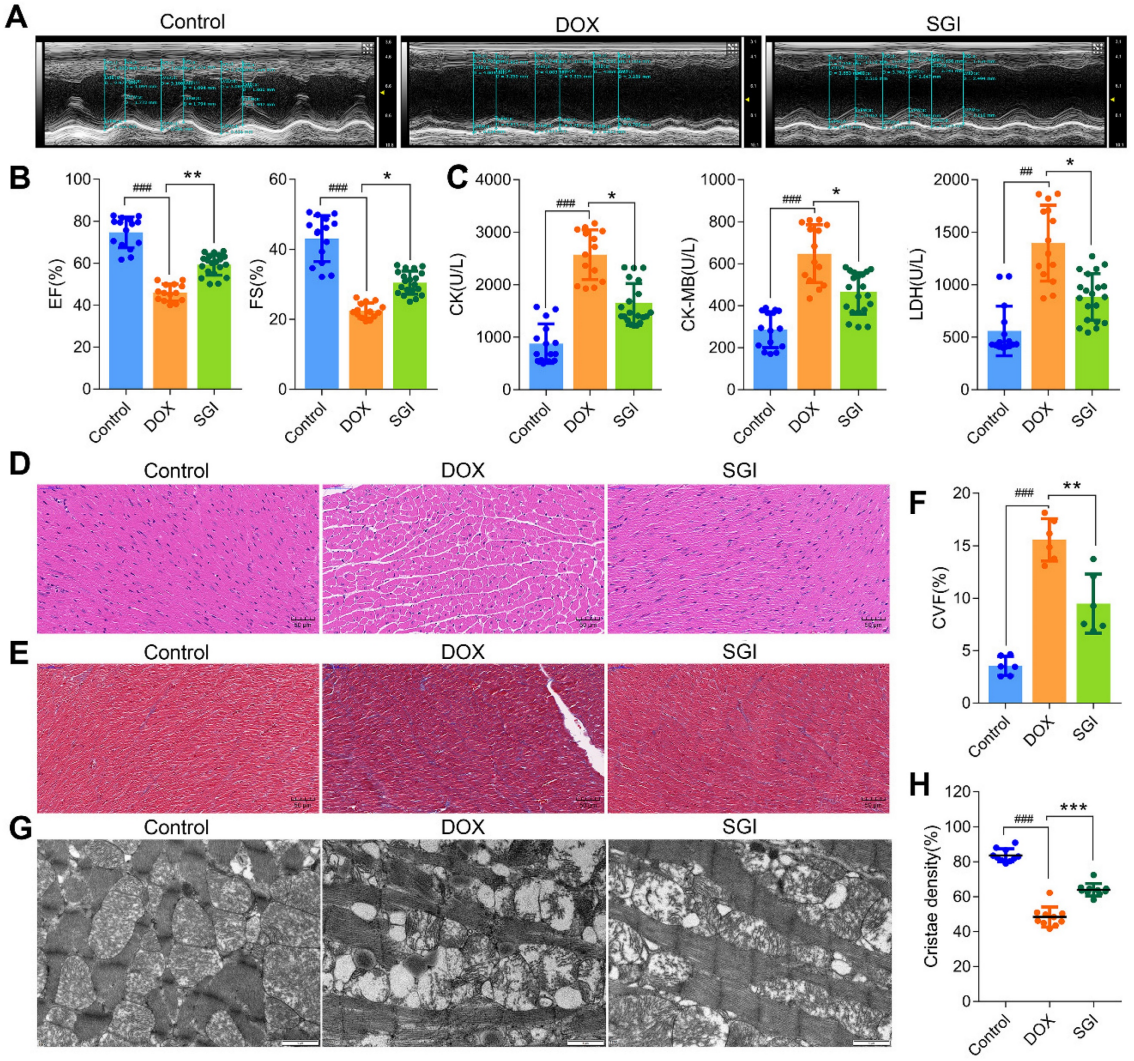
SGI combination alleviated DCM *in vivo*. (**A**) Representative two-dimensional M-mode images in each group. (**B**) Summary echocardiogram data of ejection fraction (EF%) and fractional shortening (FS%). (**C**) Contents of serum CK, CK-MB, and LDH in each group. (**D**) Representative images of H&E staining (scale bar = 50 μm, 200 ×). (**E**) Representative images of Masson (scale bar = 50 μm, 200 ×). (**F**) Collagen volume fraction (%). (**G**) Representative TEM micrographs of myocardial tissue from each group. Each image represents one individual subject (scale bar = 1 μm, 11500 ×). (**H**) Quantitative measurements of interfibrillar mitochondrial cristae density using ImageJ (=10 randomly selected images from each sample).^ ##^ *p* < 0.01, ^###^ *p* < 0.001, compared with the control group; * *p* < 0.05, ** *p* < 0.01, *** *p* < 0.001, compared with the DOX group. Data are mean ± SD, n= 6-20.

The protective effects of SGI on DCM* in vivo* were assessed in male C57BL/6 mice. The results indicated that the mortality was 30% in the DOX group, and the heart weight (HW) and body weight (BW) were significantly decreased together with a significant increase in the HW/BW ratio. After four weeks of treatment with SGI, the decreases in HW and BW were significantly ameliorated, and mortality was completely prevented (Table S2). The left ventricular ejection fraction (EF) and fractional shortening (FS) by echocardiography, reflecting left ventricular function in mice, were significantly decreased in the DOX group, which indicated that the cardiac function of mice was seriously damaged by DOX. The activities of serum CK, CK-MB and LDH (three myocardial damage marker enzymes) were significantly increased in the DOX group. As expected, SGI treatment moderated these abnormal enzymatic and functional indices in mice with DCM (Figure 6A-C).

The results from morphological analysis demonstrated that the myocardial tissues of the DOX-treated mice showed extensive cytoplasmic vacuolization and interstitial oedema as well as partial loss of nuclei, as demonstrated by H&E staining (Figure 6D), and a disordered arrangement of myocardial fibres and increased amounts of collagen deposition, as demonstrated by Masson staining (Figure 6E-F). The interfibrillar mitochondria were swollen, with disorganized and decreased cristae density (Figure 6G-H), which indicated that the cardiac structure of mice was seriously damaged. Similarly, SGI treatment effectively alleviated the above morphological changes in mice with DCM.

### UHPLC-MS-based metabolomics analysis *in vitro*

**Figure 7:**
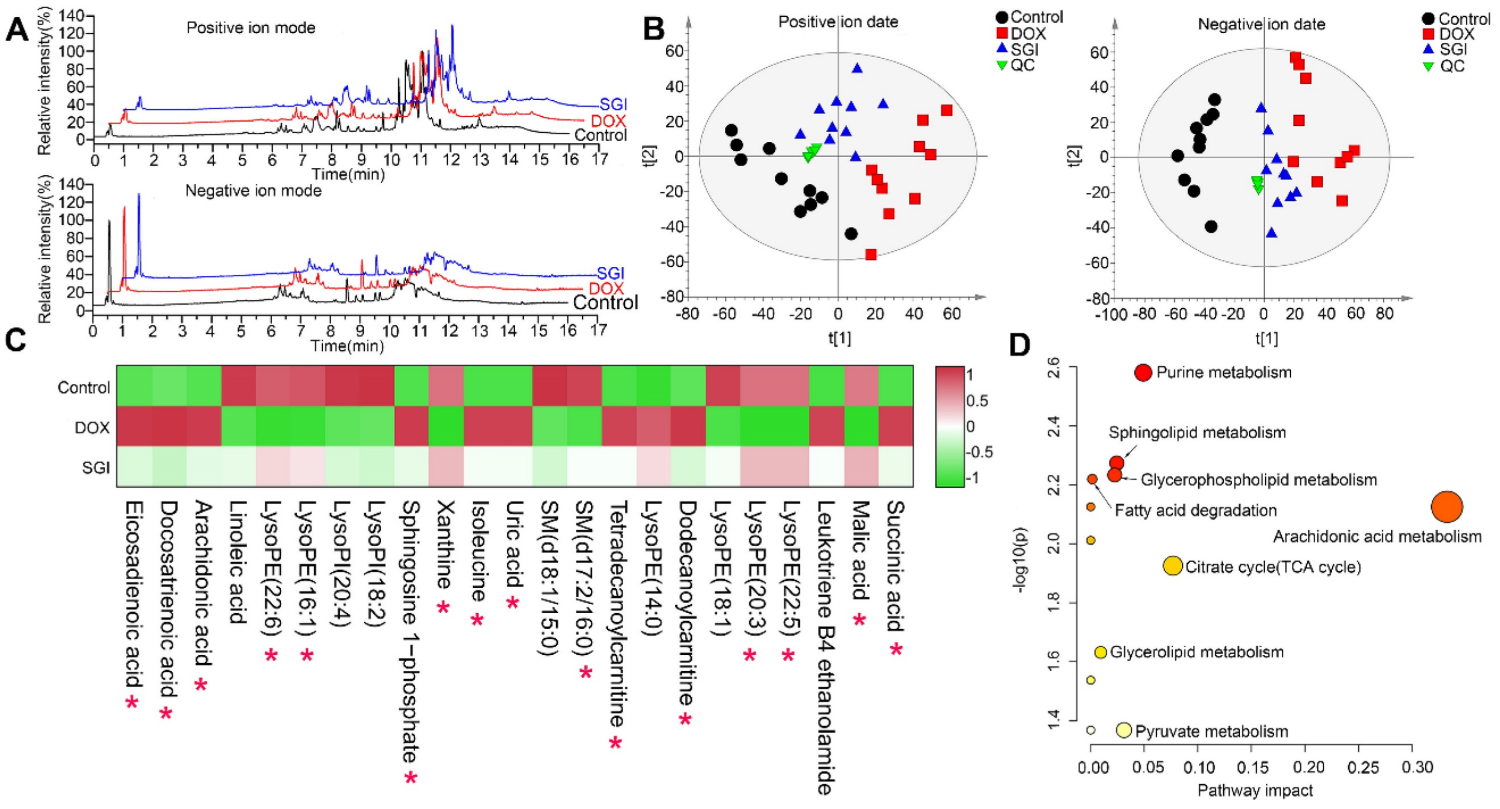
Metabolomic analyses of cardiac mitochondria samples from the control, DOX, and SGI-treated groups based on UHPLC-MS. (**A**) Representative total ion chromatogram (TIC) of the control, DOX and SGI-treated groups in the positive and negative ion modes. (**B**) PCA score plots of metabolites obtained from the positive and negative ion modes. (**C**) The heatmaps of the potential biomarkers both of control vs. DOX and SGI vs. DOX groups. The asterisk (*) indicates significant differences between the SGI-treated group and the DOX group. (**D**) The metabolic pathway enrichment analysis of SGI-reversed metabolites.

UHPLC-Q-TOFMS was employed to analyse the cardiac mitochondria samples in both positive and negative ion modes. Representative total ion chromatograms (TICs) of cardiac mitochondria samples from the control, DOX, and SGI-treated groups were obtained under optimal conditions (Figure 7A). A total of 3178 variables (ESI^+^) and 3072 variables (ESI^-^), which met the acceptance criteria with RSDs less than 30% in QCs and present in more than 75% of QCs, could be detected in 16 min, covering 87.3% and 88.3% of the respective total variables in the positive and negative LC‒MS data, respectively. The results indicated that the analytical system was stable and repeatable. Furthermore, PCA was applied to provide an overview of the real samples and QCs. As shown in Figure 7B, the QC samples clustered tightly together in the PCA score plots, which further confirmed the reliability of the analytical process. The PCA plots showed a separated tendency in the mitochondrial metabolite profiles of the control, DOX, and SGI-treated groups and demonstrated that the SGI-treated group was similar to the control group in the positive and negative ion mode data, which highlights that DOX caused mitochondrial metabolic perturbation and that SGI could neutralize the metabolic disorder in mice with DCM.

OPLS-DA was further performed to identify the metabolites that contributed to the separation of the control and DOX groups. The score plots showed apparent separation between the two groups (Figure S1A-B), and cross-validation plots generated from 200 permutation tests showed intercepts of R^2^= 0.376 and Q^2^=-0.883 in positive ion mode and R^2^= 0.248 and Q^2^= -0.547 in negative ion mode (Figure S1C-D), indicating that the respective model had good reliability and predictability. The S-plot from the OPLS model was applied to reveal the metabolites correlated with DOX-induced separation (Figure S1E-F), in which the farther a variable is away from the origin (shaded areas of Figure S1E-F), the greater the contribution to the difference between the groups and the more likely it is to be a differential metabolite. The preliminarily selected metabolites were further filtered by the criteria of VIP >1, fold change >1.5, *p* < 0.05 and false discovery rate < 0.05. Based on the MS and MS/MS spectra and the related information from the online METLIN and HMDB databases, we identified the metabolites. As listed in Table S3, a total of 23 metabolites, including 11 metabolites from the positive ion model and 12 metabolites from the negative ion model, were significantly altered in the DOX group relative to the control group. It is reasonable to take these differentially expressed metabolites (DEMs) as candidate targets for SGI treatment intervention. Therefore, these metabolites were subjected to heatmap analysis, demonstrating that SGI could reverse-regulate most of the targeted metabolites associated with DCM (Figure 7C). The ANOVA results further showed that SGI significantly reversed 16 mitochondrial metabolites (Figure S2). SGI might produce protective effects against DCM by regulating the expression of these metabolites.

Based on the KEGG database, topology and pathway enrichment analyses were performed using MetaboAnalyst 5.0 online software to identify the involved metabolomic pathways, and the pathway impact values generated from pathway topology analysis and the -log(p) from pathway enrichment analysis were calculated. Based on the values of pathway impact and -log(p), the potential pathways associated with the effect of SGI on DCM were identified and are summarized in Table S4. The identified pathways are shown in Figure 7D. The findings indicate the involvement of arachidonic acid metabolism, the TCA cycle, purine metabolism, pyruvate metabolism, sphingolipid metabolism, glycerophospholipid metabolism, glycerolipid metabolism, and fatty acid degradation.

### Label-free proteomic analysis *in vitro*

**Figure 8:**
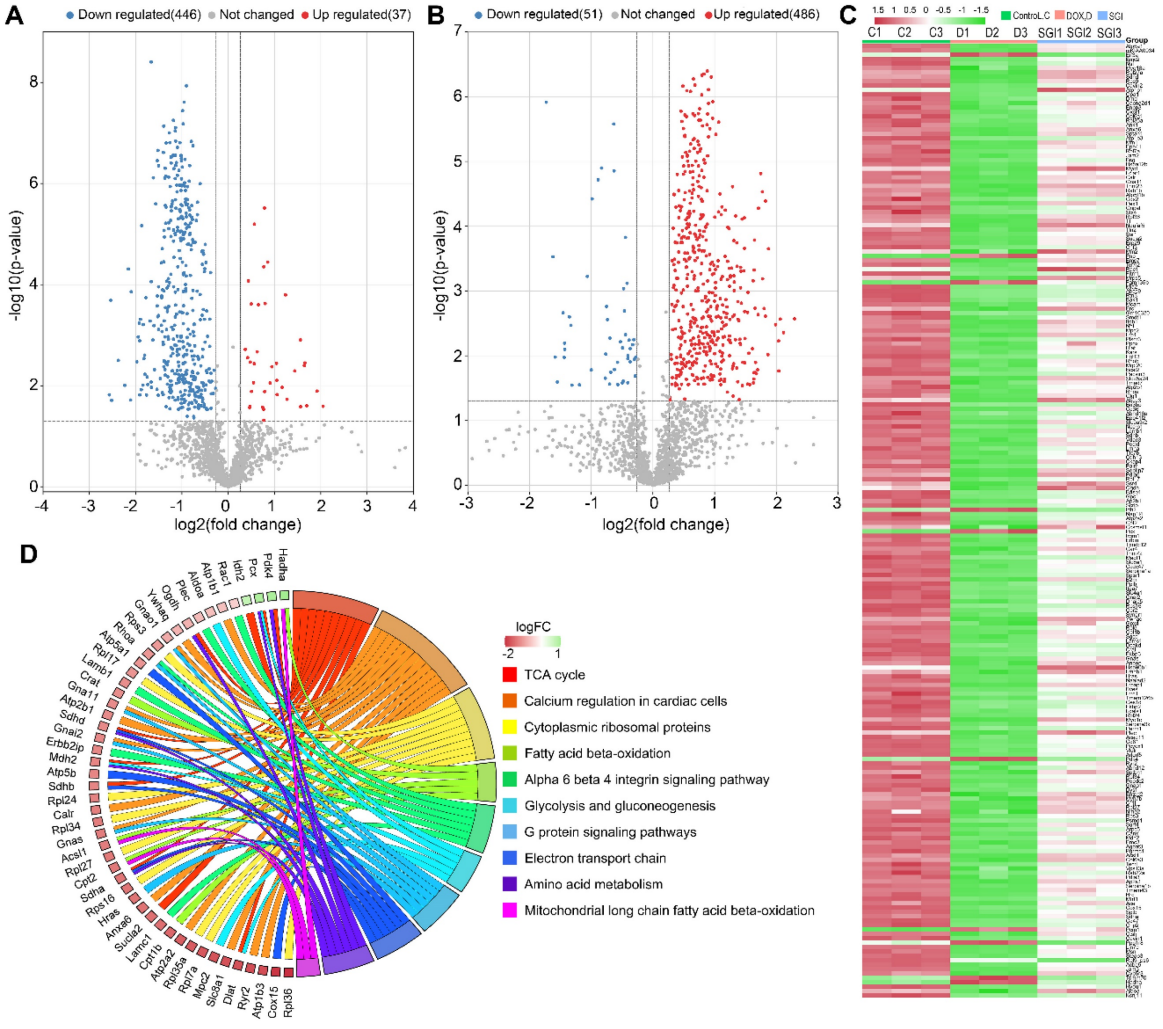
Identification, biological function and pathway enrichment analyses of DEPs. (**A** and **B**) Volcano plots of DEPs from control vs. DOX (**A**) and SGI vs. DOX (**B**). The point (blue) on the left is the protein with a downregulated expression, and the point (red) on the right is the protein with an upregulated expression. Each point in the figure represents a specific protein. (**C**) A heatmap analysis of 222 DEPs. (**D**) Chord diagram showing the top 10 enriched WikiPathways. The different colors represent the different categories to which they belong, and the color map represents fold change of proteins (log_2_FC).

To improve our understanding of the potential mechanism of SGI, cardiac mitochondrial protein profiles were investigated by label-free quantitative proteomic analysis utilizing a nano LC-MS/MS approach. As a result, 1803 proteins with FDR < 1% were identified and quantified by at least two unique peptides. Differential expression proteins (DEPs) analysis of control vs. DOX and SGI vs. DOX with the cut-offs of a Benjamini-Hochberg adjusted filter of < 0.05 and fold change >1.2, as shown in the volcano maps of Figure 8A-B, demonstrated that 483 (37 upregulated and 446 downregulated) and 537 (486 upregulated and 51 downregulated) proteins were significantly changed in the DOX group relative to the control group and the SGI-treated group relative to the DOX group, respectively. Of them, 222 overlapping DEPs between the control vs. model and SGI vs. model groups were identified (Table S5). Heatmaps of the changes in the 222 DEPs from the control, DOX, and SGI-treated groups are presented in Figure 8C.

These DEPs were categorized based on Gene Ontology (GO) annotation: biological process (BP), cellular component (CC) and molecular function (MF). The top 10 enriched GO categories for each aspect are shown in Figure S3, and some GO items such as the TCA cycle, protein binding and membrane were functionally relevant for DOX cardiotoxicity 37, 38. KEGG pathway analysis was further performed to identify the biological pathways associated with DCM, which could help to delineate the protective mechanism of SGI. As shown in Figure S4, the DEPs were involved in a variety of pathways, including metabolism, genetic information processing, environmental information processing, cellular processes, organismal systems, and human diseases, covering a wide range of biological pathways in cardiac damage. Among them, the metabolism pathways were mainly related to the TCA cycle, carbon metabolism, and fatty acid degradation. Wikipathway analysis also showed that the DEPs were involved in mitochondrial long-chain fatty acid β-oxidation, electron transport chain, calcium regulation in cardiac cells, glycolysis and gluconeogenesis, and the TCA cycle (Figure 8D). The combination of KEGG pathway analysis and Wikipathway analysis demonstrated that several pathways pertaining to energy metabolism were relevant for the cardioprotective effect of SGI, such as the TCA cycle, fatty acid β-oxidation, glycolysis and gluconeogenesis, carbon metabolism, and fatty acid metabolism, which suggested that energy metabolism plays a key role as a mediator of the cardioprotective effect of SGI in the DCM model.

### Integrated pathway analysis

**Figure 9:**
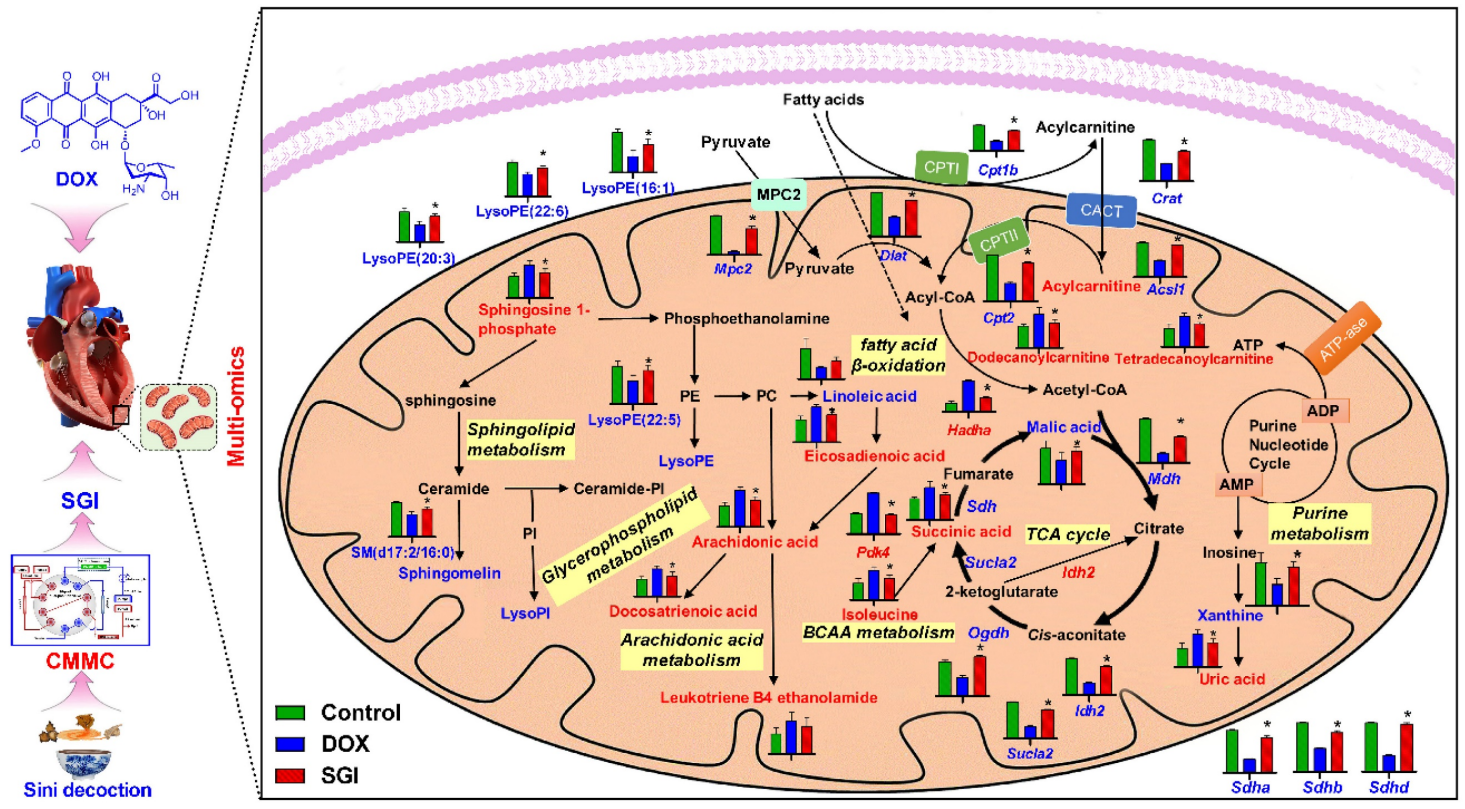
Schematic metabolic network of DCM in cardiac mitochondria and SGI modulation based on mitochondrial metabolomics and proteomics results. Column value in histograms is expressed as mean ± SD. Metabolites or proteins in red and blue represent elevation and inhibition in DOX group, respectively. Metabolites in black means they were not detected in our experiment. CMMC: cardiac mitochondrial membrane chromatography; Cpt1b, carnitine palmitoyltransferase-1B; Cpt2, Carnitine O-palmitoyltransferase 2; CACT, Carnitine-acylcarnitine translocase; Mpc2, Mitochondrial pyruvate carrier 2; Sdha, Succinate dehydrogenase [ubiquinone] flavoprotein subunit; Sdhb, Succinate dehydrogenase [ubiquinone] iron-sulfur subunit; Sdhd, Succinate dehydrogenase [ubiquinone] cytochrome b small subunit, mitochondrial; Mdh, Malate dehydrogenase, mitochondria; Sucla2, Succinate-CoA ligase [ADP-forming] subunit beta, mitochondrial; Idh2, Isocitrate dehydrogenase [NADP], mitochondrial; Ogdh, 2-oxoglutarate dehydrogenase, mitochondrial; Dlat, Dihydrolipoyllysine-residue acetyltransferase component of pyruvate dehydrogenase complex, mitochondrial; Acsl1, Long-chain-fatty-acid-CoA ligase 1; Crat, carnitine acetyltransferase; Hadha, Trifunctional enzyme subunit alpha, mitochondrial.

A total of 16 SGI-reversed DEMs and 222 DEPs were introduced to MetaboAnalyst 5.0 for joint pathway analysis. According to the integrated metabolic pathway enrichment analysis, the top three significantly different metabolic pathways were the TCA cycle, valine, leucine and isoleucine biosynthesis, and fatty acid degradation (Figure S5), suggesting a global reversed dysregulation of energy metabolism after administration of SGI. To complement the results from MetaboAnalyst, these DEMs and DEPs were submitted to IMPaLA for overrepresentation analysis. A total of 8 metabolites and 184 proteins were identified in IMPaLA, and the results were similar to those generated from MetaboAnalyst. To better understand the altered pathways, the biological relationships of the DEMs and the related proteins are summarized in Figure 9, demonstrating concordant alterations in the key proteins and their associated metabolites.

### Western blot analysis

**Figure 10:**
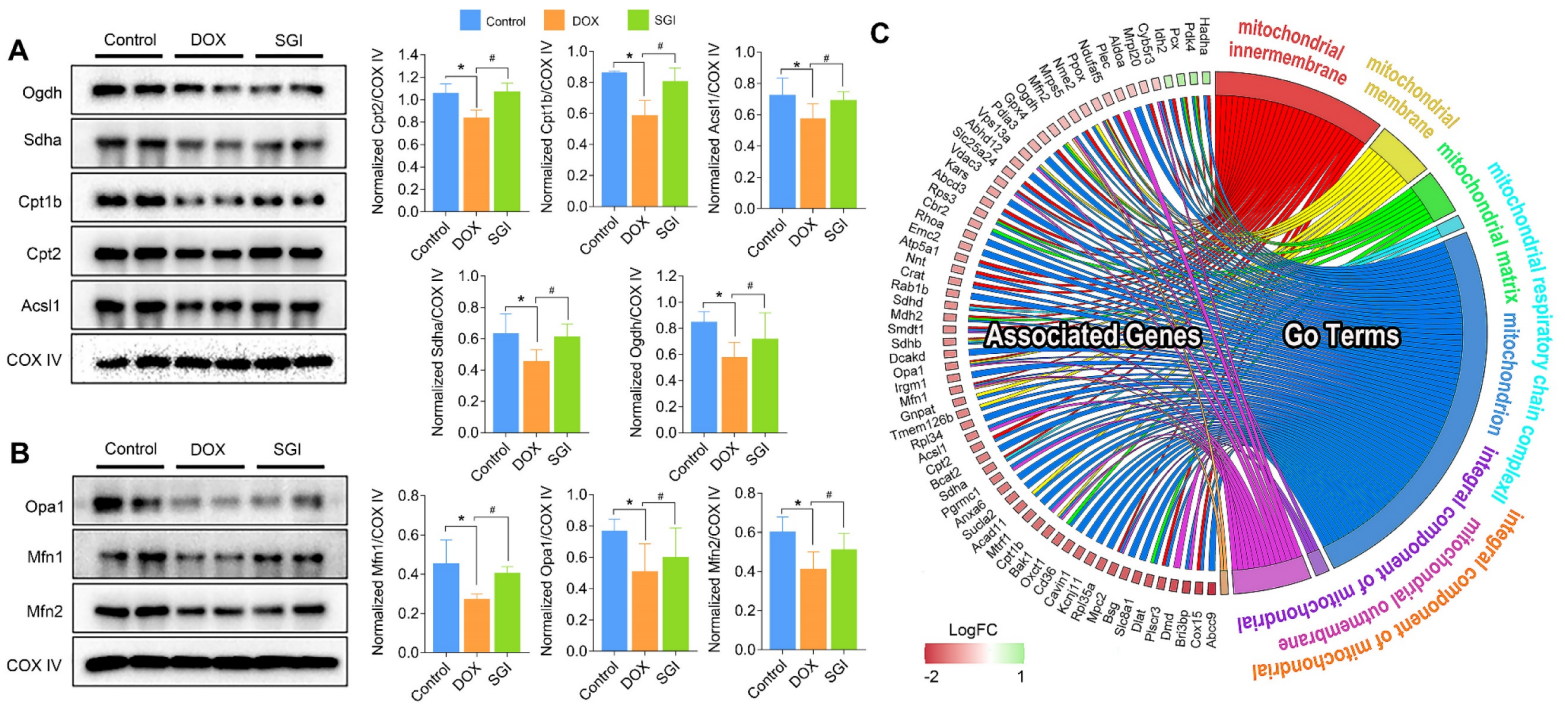
Western blotting and quantification of Ogdh, Sdha, Cpt1b, Cpt2, and Acsl1 associated with TCA cycle and fatty acid metabolism (**A**) and Opa1, Mfn1, and Mfn2 associated with mitochondrial dynamics (**B**), and (**C**) Chord plot representation of DEPs. COX IV was used as an internal control. For relative quantitation of the protein levels, band intensities were converted to arbitrary densitometric units, normalized to the value of COX IV. * *p* < 0.05, compared with the control group; ^#^ *p* < 0.05, compared with the DOX group. Data are mean ± SD, n=3. (**C**) Chord plot representation of DEPs related to mitochondria from 7 enriched pathways generated by GO terms (cellular component). The color map represents fold change of proteins (log_2_FC).

Focusing on the significantly different metabolic pathways, we used western blotting to detect and quantify the protein levels of Sdha, Acsl1, Ogdh, Cpt1b and Cpt2, which are involved in the TCA cycle and fatty acid metabolism. We also quantified the main mitochondrial dynamics-related proteins Mfn1, Mfn2 and Opa1. As a result, the protein expression levels of Sdha, Acsl1, Ogdh, Cpt1b and Cpt2 in the SGI-treated group were increased compared with those in the DOX group, which was consistent with the proteomic results (Figure 10A-B). Moreover, SGI treatment significantly upregulated the expression of Mfn1, Mfn2 and Opa1 (Figure 10C) compared with that in the DOX group, which suggested that SGI could regulate mitochondrial dynamics alterations in DCM.

## Discussion

DOX is still widely used in cancer chemotherapy, but dose-dependent cardiotoxicity severely limits its clinical use 1, 4. Several anti-DCM agents have been identified, such as carvedilol, vitamin E, and resveratrol 39-41, but these agents are far from satisfactory 42. After thousands of years of clinical practice, TCM treatments have been demonstrated to have significant efficacy and proven safety 4. One example is SND, whose efficacy in treating DCM has been well established recently. However, the cardioprotective components and mechanisms of SND against DCM are far from clear.

A large body of evidence indicates that the target organelle of DOX-induced toxicity in cardiomyocytes is mitochondria 43. The aetiology of DCM has been related to specific transporters and receptors expressed in the outer mitochondrial membrane (OMM) and inner mitochondrial membrane (IMM), and these proteins, such as TSPO and voltage-dependent calcium channels (VDAC), have become important therapeutic targets in drug development 12, 44, 45. Therefore, the cardiac mitochondria were selected as the target organelle for screening anti-DCM components from SND and dissecting the protective mechanisms at the levels of the mitochondrial proteome and metabolome.

In this study, a novel cardiac MMSP was first prepared via a mild and simple two-step aldehyde modification, in which mitochondrial membrane fragments, consisting of OMM and IMM receptors, were successfully covalently grafted onto the surface of APTES-modified silica. By using covalent immobilization, a significant increase in the immobilized mitochondrial protein content was achieved versus traditional hydrophobic interaction immobilization, indicating that the APTES-modified CMMC column offered the possibility of higher capacity. Furthermore, the lifespan of the CMMC column prepared by this covalent modification strategy was successfully prolonged from 3 to 10 days compared with the traditional unmodified column, suggesting that there was little fall-off and inactivation of mitochondrial membrane proteins during the first ten days. The selectivity results of a positive and negative drug demonstrated that the APTES-modified immobilization technique provided the ability to examine the interaction between mitochondrial membrane proteins and ligands that mimic native behaviour using the precision of CMMC. On this basis, combined with a comprehensive 2D-LC analytical system, an effective and stable 2D CMMC-TOFMS was developed to screen active components from SND targeting mitochondria to counter DCM, and S, neoline and talatizamine originating from *A. carmichaelii*, G from *Z. officinale*, and I from *G. uralensis*, which exhibited stronger retention on the first-dimension CMMC column, were screened out with protective effects against DOX cardiotoxicity in the H9c2 cell model. The cell viability results indicated that S showed higher bioactivity than neoline and talatizamine, and G was superior to I.

In the past few years, combination therapies such as multicomponent drugs for multiple targets have gained increasing attention as the next paradigm for drug discovery 35. Therefore, based on the results of pharmacological verification *in vitro* and the compatibility theory of SND 5, S, G and I were selected as a representative active ingredient combination (AIC) of* A. carmichaelii*, *Z. officinale* and *G. uralensis*, and we further focused on the efficacies and mechanisms of SGI on DCM *in vitro* and *in vivo*. In this study, *in vitro* and *in vivo* verification provided sufficient evidence to support the therapeutic effects of SGI on DCM, which further confirmed that APTES-modified CMMC has great potential as an intriguing tool for drug discovery. In addition, we showed that the combined *in vitro* use of S, G and I produced more profound therapeutic effects on cell viability, ROS generation, ATP level, and mitochondrial membrane potential than any component used individually, which supported the rationale of SND for countering DCM. Furthermore, pretreatment with SGI did not reduce the antineoplastic activity of DOX. Therefore, it was believed that SGI may be a new candidate combination to counter DCM. Moreover, we wanted to know how SGI could attenuate DCM. Therefore, an integrated mitochondrial metabolomics and proteomics approach, combined with analysis of mitochondrial structure, was applied to acquire a global perspective of the mechanism of SGI in treating DCM. To our knowledge, this is the first study that integrated mitochondrial proteomics and metabolomics analyses to investigate the pathophysiological changes of DCM and the intervention mechanism of TCM in cardiac mitochondria, a key organelle in DOX cardiotoxicity. Following the clues from the joint pathway analysis based on the DEMs and DEPs, we focused on the significant alterations in the TCA cycle, fatty acid metabolism, and branched-chain amino acid (BCAA, isoleucine, leucine and valine) metabolism in SGI-treated mice with DCM.

The TCA cycle is central to energy metabolism, facilitating adequate throughput of substrates derived from carbohydrates, fatty acids or certain amino acids 46. SGI could significantly regulate the TCA cycle intermediates malic acid and succinic acid. Beyond this, a variety of TCA cycle-related proteins, such as Idh2 (isocitrate dehydrogenase type 2), Ogdh (oxoglutarate dehydrogenase), Sucla2 (succinate-CoA ligase ADP-forming subunit β), Sdha, Sdhb, and Sdhd, were significantly regulated by SGI in the proteomics and western blot analysis.Additionally, SGI produced a significant regulatory effect on fatty acid β-oxidation-related proteins. Carnitine palmitoyltransferase I (Cpt1) and carnitine O-palmitoyltransferase 2 (Cpt2), which oxidize long-chain fatty acids in the mitochondria 47, were significantly increased in the SGI-treated group compared with the DOX group. Acsl1 (acyl-CoA synthetase long chain family member 1), which converts long-chain fatty acids to their active acyl-CoAs 48, and HADHA (hydroxyacyl-CoA dehydrogenase trifunctional multienzyme complex subunit α), which catalyses the last three steps of mitochondrial β-oxidation of long-chain fatty acids 49, were also significantly increased in the SGI-treated group compared with the DOX group. Similarly, metabolomic analyses revealed that substantial accumulations of free fatty acids (arachidonic acid, docosatrienoic acid, eicosadienoic acid) and acylcarnitine derivatives (dodecanoylcarnitine, tetradecanoylcarnitine) in the DOX group could be reversed by SGI treatment. In addition to these changes, SGI could significantly regulate BCAA (isoleucine) and BCAA metabolism-associated proteins (Pdk4, Anpep) as well as glycolysis and gluconeogenesis-associated enzymes (Mpc2, Aldoa). It seems that due to the reduction in ATP production resulting from the inhibition of fatty acid β-oxidation and the TCA cycle induced by DOX 38, SGI could partly promote the utilization of BCAAs and glycolysis as energy compensation. Taken together, these results suggest that impaired energy metabolism could be an important mechanism of functional deterioration in DCM and a potential target for SGI therapeutic intervention.

In addition, it was found that SGI could significantly regulate the metabolism of arachidonic acid, sphingomyelin (SM), lysophosphatidylinositol (LysoPI), and lysophosphatidylethanolamine (LysoPE), which are closely related to the integrity of mitochondrial membranes and mitochondrial dynamics and are also closely associated with cell inflammation and apoptosis 50-53. The results suggest that SGI critically contributed to the cardioprotective effect of DCM treatment partly via anti-inflammation and anti-apoptosis. In addition, mitochondrial morphology analysis using TEM showed reduced mitochondrial cristae in the hearts of DCM mice. The morphological changes were associated with the downregulation of key genes regulating mitochondrial membrane organization and cristae formation (Figure 10C). In particular, OPA1 (optic atrophy-1), which is a master regulator of cristae remodelling that participates in cristae junction formation and maintenance 54, was significantly downregulated in the DOX group. As expected, SGI treatment increased mitochondrial cristae density and the expression of OPA1 in mice with DCM. Therefore, Opa1-dependent mitochondrial cristae remodelling can be postulated as a therapeutic target of SGI. Opa1, in addition to its involvement in mitochondrial cristae remodelling, is a key factor implicated in mitochondrial dynamics 54. Mitochondrial dynamics play an essential role in the cellular response to DOX-induced cardiotoxicity 55. In addition to Opa1, the proteomic and western blot results demonstrated that mitofusin 1 and 2 (Mfn1 and Mfn2), which are outer mitochondrial membrane proteins involved in regulating mitochondrial dynamics 56, were significantly downregulated in the DOX group. Similarly, SGI treatment neutralized the downregulation of Mfn1 and Mfn2 in mice with DCM. It was concluded that SGI could achieve a therapeutic effect in DCM by regulating mitochondrial dynamics through promotion of the expression of Opa1, Mfn1 and Mfn2. These results indicate that SGI might be a novel candidate combination drug for the treatment of DCM and provide definitive clues on the mechanism by which SGI regulates cardiac energetics and mitochondrial dysfunction.

## Conclusions

Our study presents a novel comprehensive 2D CMMC-TOFMS analysis system for screening active components from SND to counter DCM. The CMMC column made using a mild and simple two-step aldehyde modification exhibited column lifespan extension from 3 to 10 days and excellent reproducibility when compared with the unmodified columns. Five components with potential activity against DOX cardiotoxicity, including S, neoline, talatizamine, G, and I,were screened out and confirmed by *in vitro* experiments. Based on the bioactivity evaluation and the compatibility theory of SND, S, G and I were subsequently selected as an AIC from SND. The therapeutic effects of SGI were validated *in in vitro* and* in vivo* experiments. The combined *in vitro* use of S, G and I produced more profound therapeutic effects than any component used individually on increasing ATP levels and mitochondrial membrane potential and suppressing intracellular ROS production. Moreover, an integrated model of mitochondrial metabolomics and proteomics revealed that SGI could attenuate DCM by regulating mitochondrial energy metabolism and mitochondrial dysfunction. As a whole, this work provides a scientific basis for the SGI combination in the treatment of DCM and might be considered a useful pilot trial exploring potential active components and the action mechanism of TCMs at the mitochondrial level. Importantly, the proposed covalently designed CMMC combined with the 2D-LC analytical system provides a high-throughput screening strategy for mitochondria-targeted compounds from natural products, which could be applied to other subcellular organelle models for drug discovery.

## Supplementary Material

Supplementary materials and methods, figures and tables.

## Figures and Tables

**Table 1:** Potential bioactive components in SND identified by the 2D CMMC/C18-TOFMS analysis system

No.	Identification	Retention time (min)		[M+H]^+^ m/z			Formula	Source
		CMMC	C18	Detected	Expected	error (ppm)		
5	Songorine	15.0-30.0^a^	5.6	358.2374	358.2382	-2.23	C_22_H_31_NO_3_	*A. carmichaelii*
7	Neoline	10.0-30.0^a^	5.9	438.2866	438.2856	2.28	C_24_H_39_NO_6_	*A. carmichaelii*
8	Talatizamine	10.0-22.5	6.0	422.2908	422.2906	0.47	C_24_H_39_NO_5_	*A. carmichaelii*
12	Isoliquiritigenin	2.5-22.5	7.6	257.0813	257.0814	-0.39	C_15_H_12_O_4_	*G. uralensis*
22	8-Gingerol	5.0-30.0^a^	11.7	323.2215	323.2222	-2.17	C_19_H_30_O_4_	*Z. officinale*

^a^ Not completely flushed out of the CMMC column in 30 min.

## Abbreviations

AIC: active ingredient combination
APTES: 3-aminopropyltriethoxysilane
BCA: bicinchoninic acid
CK: creatine kinase
CK-MB: creatine kinase MB
CMC: cell membrane chromatography
CMMC: cardiac mitochondrial membrane chromatography
CVF: collagen volume fraction
DCM: doxorubicin-induced cardiomyopathy
DEMs: differentially expressed metabolites
DEPs: Differentially expressed proteins
DOX: doxorubicin
EF: ejection fraction
ESI: electrospray ionization
FS: fractional shortening
HRTEM: high-resolution field emission transmission electron microscopy
IMM: inner mitochondrial membrane
LDH: lactate dehydrogenase
ΔΨm: mitochondrial membrane potential
MMSP: mitochondrial membrane stationary phase
MS: mass spectrometry
OMM: outer mitochondrial membrane
ROS: reactive oxygen species
RT: retention time
SND: Sini decoction
TCM: Traditional Chinese medicines
2D: two-dimensional
TEM: transmission electron microscopy
TOFMS: time-of-flight mass spectrometry
TSPO: translocator protein
UHPLC: ultra-high-performance liquid chromatography
VDAC: voltage-dependent calcium channels
