# Supplementary material for: Multiple mitochondria-targeted components screened from Sini decoction improved cardiac energetics and mitochondrial dysfunction to attenuate doxorubicin-induced cardiomyopathy
Source: Theranostics. 2023 Jan 1;13(2):510–30. doi: 10.7150/thno.80066 (PMC9830424; doi:10.7150/thno.80066)
Supplement: Supplementary file 1 — Supplementary materials and methods, figures and tables. [file thnov13p0510s1.pdf]

Supplementary Information

Multiple mitochondria-targeted components screened from Sini decoction improved cardiac energetics and mitochondrial dysfunction to attenuate doxorubicin-induced cardiomyopathy

Xin Ding[a,b†], Ya Zhang[a†], Pengchao Pan[c,d†], Cuiping Long[a,b], Xingxing Zhang[a,b], Lingxin Zhuo[e], Qian Zhou[f,*], Wenting Liao[e,*], Guangguo Tan[a,*]

[a] *School of Pharmacy, Air Force Medical University, Xi'an 710032, China*

[b] *School of Pharmacy, Shaanxi University of Chinese Medicine, Xi'an 712046, China*

[c] *School of Pharmacy, Naval Medical University, Shanghai 200433, China*

[d] *Department of Cardiovascular Medicine，the First Naval Hospital of Southern Theater Command, Zhanjiang 524005, China*

[e] *School of Pharmacy, China Pharmaceutical University, Nanjing 210009, China.*

[f] *Department of traditional Chinese medicine, Xijing Hospital, Air Force Medical University, Xi'an 710032, China*

[†]These authors made equal contributions to this work.

*Corresponding authors.

E-mail addresses: qianzhou2020@163.com (Qian Zhou); lwting84@163.com (Wenting Liao); guangguotan@gmail.com (Guangguo Tan).

Supplementary Materials and methods

Reagents

Three component herbs of SND, A. carmichaeli (Sichuan province), G. uralensis (Xinjiang province) and Z. officinale (Guizhou province), were obtained from Tongrentang drugstore (Xian, China). Songorine (S), 8-gingerol (G), isoliquiritigenin (I), fuziline, benzoylmesaconine, hypaconitine, neoline, talatizamine, glycyrrhizin, formononetin and 8-shogaol were purchased from Chengdu Desite Biological Technology Co., Ltd (ChengDu, China). Doxorubicin (DOX), ranitidine, succinic acid, malic acid, uric acid, isoleucine, xanthine, linoleic acid and arachidonic acid were purchased from Aladdin Industrial Company (Shanghai, China). Silica gel (5 μm, 200 Å) was purchased from Qingdao Meigao Chemical Co., Ltd. (Qingdao, China) and was activated at 120 °C before use. Dimethyl sulfoxide (DMSO), chromatography-grade acetonitrile (ACN) and formic acid were purchased from Fisher Scientific Co. (USA). (3-aminopropyl) triethoxysilane (APTES, purity 99%), 4'-chlorodiazepam, dodecanoylcarnitine, sphingosine 1-phosphate, bovine serum albumin (BSA), D-Mannitol, Percoll, HEPES, ethylene glycol bis (2-aminoethyl ether)-N-tetraacetic acid (EGTA), trizma-base and sucrose were purchased from Sigma-Aldrich (St. Louis, MO). Distilled water was purified using a Milli-Q system (Millipore, Bedford, MA, USA). Trypsin inhibitor and trypsin were purchased from Solarbio Science & Technology Co., Ltd. (Beijing, China). Lactate dehydrogenase (LDH), creatine kinase (CK) and creatine kinase MB (CK-MB) assay kits were obtained from the Nanjing Jiancheng Bioengineering Institute (Nanjing, China). An enhanced bicinchoninic acid (BCA) protein assay kit, enhanced ATP assay kit, and ROS assay kit were purchased from Beyotime Biotechnology Co., Ltd. (Shanghai, China). The following primary antibodies were used: rabbit anti-Mitofusin 2 (ab124773, Abcam, USA), rabbit anti-Sdha (ab137040, Abcam, USA), rabbit anti-Acsl1 (13989-1-AP, Proteintech, China), rabbit anti-Opa1 (27733-1-AP, Proteintech, China), rabbit anti-Ogdh (15212-1-AP, Proteintech, China), rabbit anti-Cpt1b (22170-1-AP, Proteintech, China), rabbit anti-Cpt2 (13989-1-AP, Proteintech, China), mouse anti-TSPO (68137-1-Ig, Proteintech, China), and rabbit anti-COX IV (abcam, Abcam, USA).

Transmission electron microscopy

Transmission electron microscopy was performed to confirm the integrity and purity of the purified mitochondria (PM). The fresh mitochondria pellets were fixed overnight in 2.5% glutaraldehyde in 0.01 M phosphate buffer (pH 7.4) and postfixed in 1% osmium tetroxide in 0.24 M phosphate buffer (pH 7.4). The samples were then washed with PBS buffer, dehydrated in an ethanol and graded acetone series and embedded in Epon resin. Thin sections were cut into 70 nm. The thin sections were double-stained sequentially with uranyl acetate and lead citrate prior to electron microscopy. Images were obtained using a Tecnai Spirit transmission electron microscope (Thermo fisher Scientific, USA).

Western blot analysis

Western blot analyses were performed to evaluate the purity of the prepared mitochondria. Samples were incubated for 30 min on ice with 100 µL of cell lysis buffer containing RIPA, 2 mM PMSF and protease and phosphatase inhibitor cocktail. The samples were then sonicated on ice for 30min. Next, the lysate supernatant was discarded after centrifugation at 13,000× g for 20 min, and the protein concentration was assayed using the BCA method. The protein (20 µg) was subjected to sodium dodecyl sulfate polyacrylamide gel electrophoresis (SDS-PAGE) on 10% polyacrylamide gels and then transferred onto PVDF membranes at 300 mA for 80min. Nonspecific binding was blocked with 5% skim milk in TBS-T (TBS plus 0.1% (v/v) Tween 20) at room temperature for 2 h with gentle rocking. Each membrane was then incubated with one of the following primary antibodies at 4 °C overnight: anti-COX-IV (at 1:500 dilution), anti-actin (at 1:500 dilution), anti-GAPDH (at 1:1000 dilution) and anti-cytochrome c (at 1:250 dilution) to detect mitochondrial, skeleton/plasma membrane, cytoplasm and Intermembrane space of mitochondria markers, respectively. After four washes in TBS-T, the membranes were further incubated with suitable separate secondary antibodies at room temperature for 1 h. After washed in TBST for 4 times, immunoreactive protein bands were visualized with a luminal chemiluminescence substrate using a ChemiDoc XRS image detector (Bio-Rad Laboratories Inc., Hercules, CA, USA).

Mitochondrial membrane potential (ΔΨm) measurement

5,5′,6,6′-tetrachloro-1,1′,3,3′-tetraethylbenzimidazolylcarbocyanine iodide probe (JC-1, Molecular probes) was employed to determine the mitochondrial transmembrane potential of H9c2 cells. Cells were seeded into 6-well plates and exposed to 1 μM DOX for 18 h with or without 2 h pre-treatment of 4 μg/mL SGI combination, 4 μg/mL Songorine(S), 4 μg/mL 8-gingerol(G) and 4 μg/mL isoliquiritigenin(I). Total cells were collected and incubated with an equal volume of JC-1 staining solution (5 μg/mL) at 37 °C for 20 min in the dark, and then rinsed with PBS following the manufacturer's instructions. ΔΨm was analyzed by determining the relative amounts of mitochondrial JC-1 monomers (green fluorescence, meaning lower ΔΨm) and aggregates (red fluorescence, meaning higher ΔΨm) using a fluorometer (Olympus IX53). Mitochondrial depolarization could be indicated by an increase in the green/red fluorescence intensity ratio.

Viability assay of HepG2 and K562 cells under the co-treatment of SGI and DOX

Cells were seeded at densities of 8×10^3 cells/mL for HepG2 cells and1×10^4 cells/mL for K562 cells into 96-well plates. These cells were pre-treated with different concentrations (4, 8 and 16 μg/mL) of SGI for 6h, following 2 μM DOX for 18h to make the cell injury model. Cell viability then was tested by CCK-8 (Beyotime Biotechnology, Jiangsu, China). 10 μL of CCK-8 solution was added to each well and incubated at 37 °C for 3 h. The resulting color was assayed at 450 nm using AMR-100 microplate reader (Hangzhou Allsheng Instruments Co., Ltd., China). All data were expressed as means ± standard deviation. The statistical differences were estimated with one-way ANOVA.

LC-MS-based metabolomic analysis

Data were acquired on SciexExion LC system coupled to AB SciX500R quadrupole time-of-flight (Q-TOF) mass spectrometer. Mitochondria metabolites were separated and analyzed on a Agilent Infinity LabProroshell 120 PFP column (100 × 2.1 mm, 1.9 μm) in both positive and negative ionization modes using water containing 0.1 % formic acid (v:v) as mobile phase A and 100% acetonitrile as mobile phase B. The

elution gradient and instrument parameters performed was as follows: isocratic step at 0-1 min 95%A, 1-5 min 95-50%A, 5-14 min 50-5%A, 14-16 min 5%A, 16-16.01 min, 5-95%A, 16.01-20min 95%A; flow rate, 0.4 mL/min; the column oven, 40 °C; injection volume, 2 μL; autosampler temperature, 4°C. Ion spray voltage, ±5500 V. Curtain gas and ion source gas I were set at 25, 20 psi, respectively. The collision gas was set to high. The scanning mass range of parent ion is m/z 100 ~ 1000. IDA (information dependent acquisition) sets 10 peaks with response value exceeding 10 CPS for secondary mass spectrometry scanning. The scanning range of sub ion is m/z 50 ~ 1000. Enabled dynamic background subtraction (DBS), declustering potential (DP) and collision energy (CE) for IDA detection mode were in sequence performed at 80, 10 V in TOF MS. DP, CE and CE spread were set at 80, 35,15 in TOF MS/MS, respectively.

Label-free proteomics

Proteomics experiments were performed on a Q-Exactive Plus mass spectrometer with an ancillary UltiMate 3000 HPLC system (Thermo Fisher Scientific, USA). The tryptic digested peptides were loaded on Acclaim PepMap TM 100 (15cm×75μm, nanoViper, 3 μm) C18 chromatographic column (Thermo Fisher Scientific, USA) and separated with a 70 min gradient at a flow rate of 0.35 μL/min. The column was eluted by using a gradient elution of 2% B at 0–5 min, 2%–28 %B at 5–43 min, 28%–40 %B at 43–53 min, 40 %–100 % B at 53–57 min, 100% B at 57−64 min, 100%–2%B at 57−64 min, 2% B at 65−70 min, where A was 0.1% formic acid in water and B was 80% acetonitrile modified with 0.1 % formic acid. The mass spectrometry instrument parameters were as follows: MS1 full scan resolution, 70,000; automatic gain CTR target (AGC), 1 × 106; scan range, 400 to 1500 m/z; maximum injection time (IT), 240 ms; dd-MS2 scan resolution,17,500; AGC, 1 × 105; maximum injection time, 50 ms; isolation window, 2.0 m/z; dynamic exclusion, 20.0 s; minimum AGC t, 1.00× 103. The precursor ions were fragmented by higher-energy collisional dissociation (HCD) with a normalized collision energy of 27%. Data were acquired using software provided with the instrument, and processed using Proteome

Discoverer software (Version. 2.3.0.523, Thermo Fisher Scientific, San Jose, USA).

Supplemental Tables

Table S1. Compounds of SND identified by the 2D CMMC/ C18 column-TOFMS system.

No.	Identification	Retention time (min)		$[M+H]^+$ m/z			Formula	Fragment ions (fragment voltage: 250V)	Source
		CMMC	C18	Detected	Expected	error (ppm)			
1	Chuanfunine	2.5-12.5	4.0	394.2590	394.2593	-0.76	$C_{22}H_{35}NO_5$	376.2496, 358.2375, 340.2265	AC
2	Mesaconine	2.5-10.0	4.9	486.2695	486.2703	-1.65	$C_{24}H_{39}NO_9$	406.2602, 388.2468, 374.2337	AC
3	Isotalatizidine	2.5-15.0	5.3	408.2747	408.2750	-0.73	$C_{23}H_{37}NO_5$	390.2659, 378.2561, 360.2543	AC
4	Aconine	7.5-15.0	5.5	500.2846	500.2860	-2.80	$C_{25}H_{41}NO_9$	482.2733, 468.2605, 450.2504	AC
5	Songorine	15.0-30.0[a]	5.6	358.2374	358.2382	-2.23	$C_{22}H_{31}NO_3$	340.2282, 330.2427, 312.2175	AC
6	Fuziline	2.5-15.0	5.8	454.2808	454.2805	0.66	$C_{24}H_{39}NO_7$	436.2723, 422.2559, 418.2587	AC
7	Neoline	10.0-30.0[a]	5.9	438.2866	438.2856	2.28	$C_{24}H_{39}NO_6$	420.2760, 406.2615, 388.2514	AC
8	Talatizamine	10.0-22.5	6.0	422.2908	422.2906	0.47	$C_{24}H_{39}NO_5$	390.2665, 358.2393, 326.2161	AC
9	Schaftoside	2.5-5.0	6.5	565.1553	565.1557	-0.71	$C_{26}H_{28}O_{14}$	547.1446, 529.1344, 429.0559	GU
10	14-benzoy-10-OH-mesaconine	7.5-15.0	6.8	606.2906	606.2914	-1.32	$C_{31}H_{43}NO_{11}$	588.2834, 556.2568, 542.2416	AC
11	Benzoylmesaconine	5.0-17.5	7.4	590.2986	590.2965	3.56	$C_{31}H_{43}NO_{10}$	558.2727, 526.2465, 494.2216	AC
12	Isoliquiritigenin	2.5-22.5	7.6	257.0813	257.0814	-0.39	$C_{15}H_{12}O_4$	213.0137, 137.0236	GU
13	Benzoylaconine	2.5-17.5	7.7	604.3125	604.3122	0.50	$C_{32}H_{45}NO_{10}$	586.3039, 554.2775, 540.2625	AC
14	Benzoylhypaconine	5.0-20.0	7.8	574.3017	574.3016	0.17	$C_{31}H_{43}NO_9$	542.2786, 524.2686, 492.2419	AC

15	Isoliquiritin apioside	2.5-5.0	7.9	551.1777	551.1765	2.18	$C_{26}H_{30}O_{13}$	419.1327, 257.0805, 137.0238	GU
16	Benzoyldeoxyaconine	10.0-20.0	8.0	588.3152	588.3173	-3.57	$C_{32}H_{45}NO_{9}$	556.2945, 524.2661, 496.2369	AC
17	Licoricesaponin A3	5-12.5	8.5	985.4656	985.4644	1.22	$C_{48}H_{72}O_{21}$	615.3889, 453.3405, 435.3313	GU
18	Hypaconitine	2.5-17.5	8.7	616.3143	616.3122	3.41	$C_{33}H_{45}NO_{10}$	584.2846, 538.2825, 524.2755	AC
19	Glycyrrhizin	2.5-12.5	9.9	823.4119	823.4116	0.36	$C_{42}H_{62}O_{16}$	647.3794, 471.3533, 453.3365	GU
20	Formononetin	2.5-7.5	11.2	269.0807	269.0814	-2.60	$C_{16}H_{12}O_{4}$	254.0575, 226.0618, 237.0543	GU
21	8-Shogaol	2.5-15.0	11.3	305.2113	305.2117	-1.31	$C_{19}H_{28}O_{3}$	219.6047, 137.0586	ZO
22	8-Gingerol	5.0-30.0[a]	11.7	323.2215	323.2222	-2.17	$C_{19}H_{30}O_{4}$	287.2015, 233.0483, 177.0909	ZO
23	6-Gingerol	2.5-5	12.0	295.1916	295.1909	2.37	$C_{17}H_{26}O_{4}$	233.0478, 177.0913, 162.0671	ZO
24	6-Shogaol	2.5-5	12.3	277.1799	277.1804	-1.80	$C_{17}H_{24}O_{3}$	219.6049, 137.0587	ZO

[a]Not completely flushed out of the CMMC column in 30 min. AC: Acontium carmichaeli; GU:Glycyrrhiza uralensis; ZO: Zingiber officinale.

Table S2. Effect of SGI on DOX-induced changes in heart weight (HW), body weight (BW), heart/body weight (H/BW) ratio, mortality % in C57BL/6J.

Groups	Mortality (%)	HW (g)	Initial BW (g)	Final BW (g)	HW/BW ratio (10^{-2})
Control	0	0.10±0.01	23.45±0.82	26.35±1.62	0.40±0.04
DOX	30	0.08±0.01$^{\#}$	22.97±1.11	16.86±0.96$^{\#}$	0.50±0.06$^{\#}$
SGI	0	0.08±0.01	23.00±0.59	19.33±1.40*	0.43±0.02*

Each value represents the mean ± S.D. (n=15-20). Body weight used in HW/BW ratio represents the final body weight at the end of experiment. $^{\#}$ $p < 0.05$, compared with the control group. * $p < 0.05$, compared with the DOX group.

Table S3. Identification of differentiating cardiac mitochondrial metabolic features associated with DCM.

No.	t_R	Detected m/z	Theoretical m/z	Error (ppm)	MS/MS fragment	Metabolites[a]	Formula	Adduct ion	VIP[d]	Control/DOX		SGI/DOX	
										FC[e]	P-value[f]	FC	P-value
1	0.56	119.0346	119.0344	1.34	73.0294	*Succinic acid*[b]	$C_4H_6O_4$	$[M+H]^+$	4.48	0.66	7.11E-04	0.77	1.710E-02
2	0.65	135.0292	135.0293	-0.84	89.0241, 71.0131	*Malic acid*[b]	$C_4H_6O_5$	$[M+H]^+$	2.16	1.51	8.14E-03	1.38	4.722E-02
3	6.16	380.2805	380.2800	1.30	362.2688, 344.2591, 62.0605	Leukotriene B4 ethanolamide[c]	$C_{22}H_{37}NO_4$	$[M+H]^+$	1.07	0.61	3.83E-02	0.82	6.737E-01
4	6.32	528.3112	528.3090	4.10	313.2521, 216.0645, 198.0522	*LysoPE(22:5)*[c]	$C_{27}H_{46}NO_7P$	$[M+H]^+$	1.50	1.64	8.03E-04	1.46	1.080E-02
5	6.34	504.3112	504.3090	4.35	289.2531, 216.0631, 198.0521	*LysoPE(20:3)*[c]	$C_{25}H_{46}NO_7P$	$[M+H]^+$	1.81	1.75	1.22E-05	1.51	7.662E-04
6	6.50	480.3111	480.3090	4.47	265.2533, 216.0629, 198.0531	LysoPE(18:1)[c]	$C_{23}H_{46}NO_7P$	$[M+H]^+$	3.66	1.61	2.93E-04	1.23	1.898E-01
7	6.63	344.2812	344.2800	3.54	285.2091, 144.1011, 85.0287	*Dodecanoylcarnitine*[b]	$C_{19}H_{37}NO_4$	$[M+H]^+$	1.05	0.64	8.23E-04	0.74	1.111E-02
8	7.40	426.2616	426.2620	-1.05	211.2064, 216.0638, 198.0529	LysoPE(14:0)[c]	$C_{19}H_{40}NO_7P$	$[M+H]^+$	1.07	0.66	1.60E-03	0.86	2.969E-01
9	8.37	372.3131	372.3113	4.76	313.2377, 85.0282	*Tetradecanoylcarnitine*[c]	$C_{21}H_{41}NO_4$	$[M+H]^+$	1.23	0.60	4.41E-05	0.76	5.335E-03
10	9.56	687.5472	687.5441	4.51	184.0737, 104.1069, 60.0804	*SM(d17:2/16:0)*[c]	$C_{38}H_{75}N_2O_6P$	$[M+H]^+$	1.24	1.53	1.18E-06	1.24	4.125E-03
11	9.64	689.5627	689.5597	4.41	184.0733, 104.1073, 60.0808	SM(d18:1/15:0)[c]	$C_{38}H_{77}N_2O_6P$	$[M+H]^+$	1.03	1.52	6.03E-06	1.15	1.626E-01
12	0.64	167.0206	167.0205	0.81	124.0157	*Uric acid*[b]	$C_5H_4N_4O_3$	$[M-H]^-$	1.31	0.56	1.38E-04	0.74	1.216E-02
13	0.68	130.0865	130.0868	-2.00	67.2136	*Isoleucine*[b]	$C_6H_{13}NO_2$	$[M-H]^-$	1.76	0.56	1.38E-04	0.74	1.216E-02
14	0.69	151.0250	151.0256	-4.02	108.0224, 65.9988	*Xanthine*[b]	$C_5H_4N_4O_2$	$[M-H]^-$	1.43	1.99	1.37E-04	1.79	1.210E-03
15	5.38	378.2411	378.2409	0.44	78.9597	*Sphingosine 1-phosphate*[b]	$C_{18}H_{38}NO_5P$	$[M-H]^-$	1.16	0.65	1.36E-03	0.77	2.690E-02
16	6.11	595.2911	595.2883	4.74	433.2364, 279.2335, 152.9961	LysoPI(18:2)[c]	$C_{27}H_{49}O_{12}P$	$[M-H]^-$	2.21	2.01	7.45E-05	1.26	4.339E-01
17	6.15	619.2914	619.2883	4.94	457.2369, 303.2341, 285.2227	LysoPI(20:4)[c]	$C_{29}H_{49}O_{12}P$	$[M-H]^-$	8.25	2.58	4.33E-09	1.30	7.537E-02
18	6.21	450.2635	450.2621	3.16	253.2177, 235.2064, 78.9597	*LysoPE(16:1)*[c]	$C_{21}H_{42}NO_7P$	$[M-H]^-$	1.10	2.52	4.43E-06	1.74	5.882E-03

19	6.31	524.2802	524.2777	4.69	327.2337, 309.2229, 78.9595	*LysoPE(22:6)*[c]	$C_{27}H_{44}NO_7P$	$[M-H]^-$	12.07	1.52	2.13E-06	1.29	1.166E-03
20	8.59	279.2333	279.2324	3.20	261.0209, 59.0136	Linoleic acid[b]	$C_{18}H_{32}O_2$	$[M-H]^-$	9.29	2.57	5.32E-04	1.52	3.627E-01
21	8.60	303.2331	303.2324	2.36	231.2105, 177.1634	*Arachidonic acid*[b]	$C_{20}H_{32}O_2$	$[M-H]^-$	9.69	0.58	1.32E-05	0.72	9.127E-04
22	9.20	333.2788	333.2794	-1.88	287.2748, 59.0132	*Docosatrienoic acid*[c]	$C_{22}H_{38}O_2$	$[M-H]^-$	1.00	0.63	2.96E-04	0.74	7.154E-03
23	9.33	307.2648	307.2637	3.61	289.2536	*Eicosadienoic acid*[c]	$C_{20}H_{36}O_2$	$[M-H]^-$	1.21	0.64	2.93E-04	0.78	2.016E-02

[a] The metabolites marked with "[b]" were structurally identified by reference standards, the metabolites marked with "[c]" were putatively annotated, and those in italics were significantly reversed metabolites by SGI. [d] Variable importance in the projection (VIP) was obtained from the OPLS-DA model. [e] The FC (fold change) was calculated from the average mass response (normalized peak area) of control group vs. DOX group and SGI-treated group vs. DOX group. [f] The p value was calculated from ANOVA. Abbreviations: LysoPE, lysophosphatidylethanolamine; LysoPC, Lysophosphatidylcholine; LysoPI, lysophosphatidylinositol; SM, sphingomyelin.

Table S4. Metabolic pathways associated to varied influences of SGI in DCM.

	Total[a]	Hits[b]	-log (p)[c]	FDR	Impact[d]
Arachidonic acid metabolism	36	1	2.1254	0.0173	0.3329
Citrate cycle (TCA cycle)	20	2	1.9255	0.0190	0.0769
Purine metabolism	66	2	2.5805	0.0173	0.0491
Pyruvate metabolism	22	1	1.3687	0.0428	0.0311
Sphingolipid metabolism	21	2	2.2738	0.0173	0.0243
Glycerophospholipid metabolism	36	2	2.2333	0.0173	0.0225
Glycerolipid metabolism	16	1	1.6309	0.0332	0.0094
Fatty acid degradation	39	2	2.2195	0.0173	0.0014

[a] Total is the total number of compounds in the pathway; [b] the hits is the actually matched number from the user uploaded data; [c] the p is the original p value calculated from the enrichment analysis; [d] the impact is the pathway impact value calculated from pathway topology analysis.

Table S5. The differential protein changes of cardiac mitochondria associated with SGI-reversed in mice.

Accession	Description	Gene	DOX/Control		SGI/DOX	
			FC [a]	*p*-value [b]	FC [a]	*p*-value [b]
D3Z6F5	ATP synthase subunit alpha	Atp5a1	0.56	0.00000	1.47	0.00001
Q80U89	MKIAA0034 protein (Fragment)	mKIAA0034	0.49	0.00001	1.65	0.00000
P23116	Eukaryotic translation initiation factor 3 subunit A	Eif3a	1.48	0.00367	0.51	0.00004
Q9CRD2	ER membrane protein complex subunit 2	Emc2	0.56	0.00000	1.34	0.00007
P46460	Vesicle-fusing ATPase	Nsf	0.56	0.00001	1.24	0.00162
E9Q405	Unconventional myosin-XVIIIa	Myo18a	0.57	0.00096	1.51	0.00411
P14115	60S ribosomal protein L27a	Rpl27a	0.59	0.00001	1.68	0.00002
Q9CXV1	Succinate dehydrogenase [ubiquinone] cytochrome b small subunit, mitochondrial	Sdhd	0.53	0.00000	1.84	0.00003
Q6ZWN5	40S ribosomal protein S9	Rps9	0.54	0.00000	1.59	0.00000
Q63918	Caveolae-associated protein 2	Cavin2	0.43	0.00000	1.58	0.00022
Q3TV47	Sodium/potassium-transporting ATPase subunit beta	Atp1b1	0.71	0.00002	2.03	0.00000
P58281-2	Isoform 2 of Dynamin-like 120 kDa protein, mitochondrial	Opa1	0.50	0.00000	1.39	0.00012
P11531	Dystrophin	Dmd	0.37	0.00017	1.66	0.00100
O08532-4	Isoform 2D of Voltage-dependent calcium channel subunit alpha-2/delta-1	Cacna2d1	0.47	0.00004	1.76	0.00078
Q6DYE8	Ectonucleotide pyrophosphatase/phosphodiesterase family member 3	Enpp3	0.54	0.00038	1.42	0.00449
Q9D0K2	Succinyl-CoA:3-ketoacid coenzyme A transferase 1, mitochondrial	Oxct1	0.43	0.00000	1.68	0.00002
Q04857	Collagen alpha-1(VI) chain	Col6a1	0.46	0.00025	1.50	0.00032
Q6ZWX1	Ribosomal protein L35A	Rpl35a	0.41	0.00001	1.60	0.00013
E9QNT8	Ankyrin-1	Ank1	0.40	0.00009	1.82	0.00016
P14824	Annexin A6	Anxa6	0.46	0.00000	1.84	0.00009
E9Q447	Spectrin alpha chain, non-erythrocytic 1	Sptan1	0.41	0.00011	2.09	0.00006
P97370	Sodium/potassium-transporting ATPase subunit beta-3	Atp1b3	0.38	0.00000	1.39	0.00001
Q811U4	Mitofusin-1	Mfn1	0.50	0.00001	1.69	0.00012
A2A842	Protein 4.1	Epb41	0.43	0.00177	1.86	0.00140
Q58ET1	Ribosomal protein L7A	Rpl7a	0.40	0.00012	1.67	0.00000
Q9CWD9	Junctional adhesion molecule B	Jam2	0.58	0.00000	1.42	0.00004
K3W4Q8	Basigin	Bsg	0.39	0.00000	1.58	0.00022
Q9CZJ2	Heat shock 70 kDa protein 12B	Hspa12b	0.52	0.00000	1.52	0.00001
A0A286YDF5	Myoferlin	Myof	0.61	0.00001	1.91	0.00038
B0V2Q7	Lclat1 protein	Lclat1	0.53	0.00004	1.48	0.00055
B2MWM9	Calreticulin	Calr	0.49	0.00001	1.74	0.00000
Q3UPA1	Uncharacterized protein	Gna11	0.53	0.00000	1.52	0.00006
Q8BGX0	E3 ubiquitin-protein ligase TRIM23	Trim23	0.54	0.00000	1.85	0.00000
Q9D1G1	Ras-related protein Rab-1B	Rab1b	0.53	0.00001	1.67	0.00001
P56480	ATP synthase subunit beta, mitochondrial	Atp5f1b	0.51	0.00014	1.73	0.00007
Q99L43	Phosphatidate cytidylyltransferase 2	Cds2	0.53	0.00603	1.28	0.00048
Q5BL07	Peroxisome biogenesis factor 1	Pex1	0.50	0.00000	1.77	0.00002
Q8R390	Dihydroxyacetone phosphate acyltransferase	Gnpat	0.49	0.00000	1.39	0.00018
P70452	Syntaxin-4	Stx4	0.48	0.00035	1.36	0.00006
Q6ZWZ4	60S ribosomal protein L36	Rpl36	0.34	0.00000	2.45	0.00002
Q921I1	Serotransferrin	Tf	0.53	0.00000	1.73	0.00001
A2APY7	Arginine-hydroxylase NDUFAF5, mitochondrial	Ndufaf5	0.68	0.00002	1.65	0.00001
A0A1L1SQ51	Talin-2	Tln2	0.44	0.00011	1.82	0.00029
A0A286YDB7	Translocon-associated protein subunit alpha (Fragment)	Ssr1	0.46	0.00000	1.58	0.00000
Q9Z2I9	Succinate--CoA ligase [ADP-forming] subunit beta, mitochondrial	Sucla2	0.46	0.00000	1.71	0.00001

P57759	Endoplasmic reticulum resident protein 29	Erp29	0.50	0.00000	1.62	0.00001
Q9Z2L7	Cytokine receptor-like factor 3	Crlf3	0.33	0.00000	1.70	0.00001
Q80U63	Mitofusin-2	Mfn2	0.67	0.02493	1.74	0.00265
Q543K9	Purine nucleoside phosphorylase	Pnp	2.36	0.00016	0.77	0.00573
Q9EP72	ER membrane protein complex subunit 7	Emc7	0.52	0.00000	1.69	0.00000
Q3V1G9	HMG box domain-containing protein	Tcf7l2	0.63	0.00000	1.27	0.00003
Q9D859	Uncharacterized protein	Rac1	0.77	0.00006	1.71	0.00007
Q91V79	Fat storage-inducing transmembrane protein 1	Fitm1	0.35	0.00000	1.51	0.00007
Q3U2U4	S5 DRBM domain-containing protein	Mrps5	0.67	0.00002	1.58	0.00003
Q8BLS3	Family with sequence similarity 135, member B	Fam135b	1.57	0.00025	0.81	0.00562
H3BKL6	Melanoma inhibitory activity protein 2	Mia2	0.38	0.00013	1.51	0.00030
E9PUE8	ATP-binding cassette sub-family C member 9	Abcc9	0.32	0.00000	1.52	0.00004
Q9ES97	Reticulon-3	Rtn3	0.22	0.00005	1.86	0.00244
O08734	Bcl-2 homologous antagonist/killer	Bak1	0.43	0.00000	1.47	0.00009
Q8R2Y2	Cell surface glycoprotein MUC18	Mcam	0.47	0.00001	1.68	0.00023
F8VPM7	ELKS/Rab6-interacting/CAST family member 1	Erc1	0.70	0.00006	1.48	0.00001
E9PW43	Protein transport protein Sec61 subunit beta	Gm10320	0.45	0.00001	1.25	0.00395
Q9DB10	Essential MCU regulator, mitochondrial	Smdt1	0.51	0.00006	1.51	0.00009
P09055	Integrin beta-1	Itgb1	0.49	0.00000	1.45	0.00043
Q61941	NAD(P) transhydrogenase, mitochondrial	Nnt	0.55	0.00001	1.56	0.00001
Q9D023	Mitochondrial pyruvate carrier 2	Mpc2	0.39	0.00000	1.81	0.00873
A0A0R4J0I9	Prolow-density lipoprotein receptor-related protein 1	Lrp1	0.46	0.00000	1.96	0.00000
Q3T9B7	Phospholipid scramblase	Plscr3	0.38	0.00001	1.57	0.00002
A0A0A6YX50	Protoporphyrinogen oxidase (Fragment)	Ppox	0.68	0.00004	1.33	0.00520
Q8BMF4	Dihydrolipoyllysine-residue acetyltransferase component of pyruvate dehydrogenase complex, mitochondrial	Dlat	0.39	0.00000	1.89	0.00000
Q8CFK5	Lysine--tRNA ligase	Kars	0.58	0.00000	1.34	0.00014
P70377	Fibroblast growth factor 13	Fgf13	0.44	0.00000	1.34	0.00003
P62746	Rho-related GTP-binding protein RhoB	Rhob	0.51	0.00001	1.57	0.00001
V9GXI7	39S ribosomal protein L20, mitochondrial	Mrpl20	0.70	0.00005	1.27	0.00082
A0A1B0GX27	Branched-chain-amino-acid aminotransferase	Bcat2	0.47	0.00001	1.47	0.00026
Q543N7	Uncharacterized protein	Pacsin3	0.52	0.00000	1.28	0.00174
Q8BMD8	Calcium-binding mitochondrial carrier protein SCaMC-1	Slc25a24	0.59	0.00000	1.60	0.00000
E9Q7G1	Transmembrane p24-trafficking protein 7	Tmed7	0.68	0.00040	1.30	0.00064
G5E829	Plasma membrane calcium-transporting ATPase 1	Atp2b1	0.53	0.00010	1.44	0.00003
Q9QUI0	Transforming protein RhoA	Rhoa	0.56	0.00000	1.43	0.00001
F8WHM5	Golgi apparatus protein 1 (Fragment)	Glg1	0.47	0.00342	1.71	0.00000
Q3U645	Uncharacterized protein	Abcd3	0.58	0.00205	2.00	0.00144
Q8BXV2	BRI3-binding protein	Bri3bp	0.35	0.00000	1.33	0.00036
Q08857	Platelet glycoprotein 4	Cd36	0.43	0.00000	1.84	0.00000
Q9Z1Q2	Phosphatidylserine lipase ABHD16A	Abhd16a	0.53	0.00014	1.23	0.00960
O70318	Band 4.1-like protein 2	Epb41l2	0.35	0.00017	1.79	0.00526
Q9JHL1	Na(+)/H(+) exchange regulatory cofactor NHE-RF2	Slc9a3r2	0.39	0.00000	2.09	0.00000
G3X9C2	F-box only protein 50	Nccrp1	0.54	0.00002	1.27	0.00039
B9EKB0	Laminin B1 subunit 1	Lamb1	0.53	0.00000	1.57	0.00000
Q9CQA3	Succinate dehydrogenase [ubiquinone] iron-sulfur subunit, mitochondrial	Sdhb	0.51	0.00000	1.51	0.00858
J3QMG3	Voltage-dependent anion-selective channel protein 3	Vdac3	0.59	0.00013	1.41	0.00096
Q3UYF4	Uncharacterized protein (Fragment)	Podxl	0.41	0.00000	1.95	0.00000
P48678	Prelamin-A/C	Lmna	0.37	0.00004	1.70	0.00013
A0A1Y7VL44	Tetratricopeptide repeat protein 7B	Ttc7b	0.60	0.00103	1.38	0.00108

Q8VDK4	Cadherin 13	Cdh13	0.50	0.00000	1.51	0.00001
Q8BMK4	Cytoskeleton-associated protein 4	Ckap4	0.53	0.00007	1.67	0.00003
Q6ZQE7	MKIAA0270 protein (Fragment)	Palm	0.45	0.00000	1.47	0.00001
E9Q1G8	Septin-7	Septin7	0.48	0.00000	1.87	0.00000
Q3TML0	Protein disulfide-isomerase A6	Pdia6	0.57	0.00002	1.77	0.00000
Q80V08	Rpl17 protein (Fragment)	Rpl17	0.55	0.00000	1.34	0.00005
Q9D8L3	Signal sequence receptor, delta	Ssr4	0.58	0.00019	1.81	0.00010
Q60597	2-oxoglutarate dehydrogenase, mitochondrial	Ogdh	0.66	0.00049	1.73	0.00024
Q91XV3	Brain acid soluble protein 1	Basp1	0.43	0.00000	1.44	0.00001
P09055-2	Isoform 2 of Integrin beta-1	Itgb1	0.62	0.00001	1.50	0.00007
Q9QZF2	Glypican-1	Gpc1	0.42	0.00000	1.47	0.00015
Q5SWR1	AP complex subunit beta	Ap2b1	0.55	0.00002	1.49	0.00010
Q6P9N3	Scrib protein	Scrib	0.41	0.00120	1.53	0.00579
P54071	Isocitrate dehydrogenase [NADP], mitochondrial	Idh2	1.48	0.00001	0.64	0.00000
Q8C1W9	Uncharacterized protein	Nap1l4	0.41	0.00334	1.46	0.00000
O55143	Sarcoplasmic/endoplasmic reticulum calcium ATPase 2	Atp2a2	0.41	0.00000	1.57	0.00020
P52825	Carnitine O-palmitoyltransferase 2	Cpt2	0.48	0.00000	1.72	0.00003
Q6RUT7	Protein CCSMST1	Ccsmst1	0.77	0.00860	1.37	0.00974
G5E8R3	Pyruvate carboxylase	Pcx	1.71	0.00004	0.75	0.00076
Q60766	Immunity-related GTPase family M protein 1	Irgm1	0.50	0.00000	1.41	0.00002
O08532-3	Isoform 2C of Voltage-dependent calcium channel subunit alpha-2/delta-1	Cacna2d1	0.49	0.00000	1.74	0.00004
B2RUJ2	Erbb2ip protein	Erbin	0.51	0.00006	1.59	0.00025
Q9CQU0	Thioredoxin domain-containing protein 12	Txndc12	0.53	0.00000	1.40	0.00000
Q3U3I1	Alpha-carbonic anhydrase domain-containing protein	Car4	0.64	0.00300	1.43	0.00032
Q1XH17	Tripartite motif-containing protein 72	Trim72	0.48	0.00002	1.64	0.00016
A2ADH1	Magnesium transporter protein 1	Magt1	0.44	0.00077	1.29	0.00005
G3X9J1	Sodium/calcium exchanger 1	Slc8a1	0.39	0.00000	1.78	0.00024
Q9D024	Coiled-coil domain-containing protein 47	Ccdc47	0.51	0.00001	1.49	0.00006
P07758	Alpha-1-antitrypsin 1-1	Serpina1a	0.44	0.00001	1.57	0.00000
P08032	Spectrin alpha chain, erythrocytic 1	Spta1	0.38	0.00001	1.55	0.00004
Q3U679	Beta-2-microglobulin	B2m	0.50	0.00000	1.73	0.00000
P24369	Peptidyl-prolyl cis-trans isomerase B	Ppib	0.39	0.00000	1.45	0.00035
G3X9Q1	Integrin alpha-7	Itga7	0.38	0.00138	1.53	0.00296
Q3TNL6	Anion exchange protein	Slc4a1	0.43	0.00000	1.51	0.00003
P08752	Guanine nucleotide-binding protein G(i) subunit alpha-2	Gnai2	0.52	0.00001	1.27	0.00032
P60904	DnaJ homolog subfamily C member 5	Dnajc5	0.58	0.00002	1.43	0.00026
Q641N3	Rps16 protein (Fragment)	Rps16	0.47	0.00000	1.24	0.00034
Q542P5	Uncharacterized protein	Cbr2	0.57	0.00007	1.31	0.00018
O55100	Synaptogyrin-1	Syngr1	0.57	0.00001	1.37	0.00005
A3KML3	Tyrosine 3-monooxygenase/tryptophan 5-monooxygenase activation protein, theta polypeptide	Ywhaq	0.66	0.00000	1.53	0.00000
O70325	Phospholipid hydroperoxide glutathione peroxidase	Gpx4	0.66	0.00016	1.37	0.00367
Q3THE1	Ribosomal_L18_c domain-containing protein (Fragment)	Rpl5	0.46	0.00000	1.52	0.00013
Q924X2	Carnitine O-palmitoyltransferase 1	Cpt1b	0.43	0.00000	1.74	0.00007
Q544D4	Sarcoglycan, delta (Dystrophin-associated glycoprotein)	Sgcd	0.52	0.00001	1.58	0.00006
F8VQJ3	Laminin subunit gamma-1	Lamc1	0.45	0.00001	1.61	0.00014
Q8BHC4	Dephospho-CoA kinase domain-containing protein	Dcakd	0.50	0.00000	1.30	0.00097
H7BX88	Carnitine O-acetyltransferase	Crat	0.53	0.00000	1.38	0.00015
Q62446	Peptidyl-prolyl cis-trans isomerase FKBP3	Fkbp3	0.56	0.00000	1.26	0.00003
P63094	Guanine nucleotide-binding protein G(s) subunit alpha isoforms short	Gnas	0.49	0.00000	1.68	0.00015

P97449	Aminopeptidase N	Anpep	0.51	0.00006	1.73	0.00038
Q91V38	Heat shock protein 90, beta (Grp94), member 1	Hsp90b1	0.69	0.00003	1.87	0.00000
P14733	Lamin-B1	Lmnb1	0.69	0.00031	1.49	0.00010
Q61411	GTPase HRas	Hras	0.46	0.00211	1.60	0.00000
Q3UZV6	N-acetylated alpha-linked acidic dipeptidase 2	Naalad2	0.54	0.00000	1.42	0.00015
Q52KI7	Low density lipoprotein receptor-related protein associated protein 1	Lrpap1	0.45	0.00000	1.93	0.00000
Q9ES83	Blood vessel epicardial substance	Bves	0.45	0.00000	1.53	0.00001
Q80VQ1	Leucine-rich repeat-containing protein 1	Lrrc1	0.49	0.00035	1.29	0.00018
Q9D1R1	Complex I assembly factor TMEM126B, mitochondrial	Tmem126b	0.49	0.00001	1.60	0.00058
O55143-2	Isoform 2 of Sarcoplasmic/endoplasmic reticulum calcium ATPase 2	Atp2a2	0.43	0.00805	1.46	0.00043
Q8VCT4	Carboxylesterase 1D	Ces1d	0.38	0.00000	1.41	0.00014
P45878	Peptidyl-prolyl cis-trans isomerase FKBP2	Fkbp2	0.50	0.00004	1.28	0.00384
P16045	Galectin-1	Lgals1	0.41	0.00001	1.62	0.00014
Q3UW40	TRASH domain-containing protein	Rpl24	0.50	0.00000	1.43	0.00000
Q9WTI7	Unconventional myosin-Ic	Myo1c	0.53	0.00346	1.85	0.00001
A0A0R4J0I1	Serine protease inhibitor A3K	Serpina3k	0.39	0.00001	1.81	0.00010
Q149B8	PGC-1 and ERR-induced regulator in muscle protein 1	Perm1	0.51	0.00001	1.26	0.00203
Q6S390	Plectin 4	Plec	0.69	0.00002	1.61	0.00010
A0A0R4J0I6	Acyl-CoA dehydrogenase family member 11	Acad11	0.44	0.00003	1.61	0.00034
Q3UWG5	Tetraspanin	Cd81	0.49	0.00000	1.49	0.00001
Q9CQF9	Prenylcysteine oxidase	Pcyox1	0.42	0.00000	1.55	0.00003
F8WGI9	Very low-density lipoprotein receptor	Vldlr	0.46	0.00038	1.57	0.00024
A0A286YDT5	Adhesion G protein-coupled receptor F5 (Fragment)	Adgrf5	0.45	0.00000	1.55	0.00660
Q544J2	Protein-serine/threonine kinase	Pdk4	1.72	0.00000	0.56	0.00001
E9Q401	Ryanodine receptor 2	Ryr2	0.38	0.00004	2.00	0.00007
D6RFU2	Lysophosphatidylserine lipase ABHD12	Abhd12	0.60	0.00258	1.28	0.01320
Q62261	Spectrin beta chain, non-erythrocytic 1	Sptbn1	0.45	0.00014	1.98	0.00040
Q9D1R9	60S ribosomal protein L34	Rpl34	0.49	0.00497	1.46	0.00000
Q9ES82	Popeye domain-containing protein 2	Popdc2	0.43	0.00000	1.77	0.00001
P18872	Guanine nucleotide-binding protein G(o) subunit alpha	Gnao1	0.63	0.00000	1.26	0.00011
A0A0N4SWH3	Dysferlin	Dysf	0.37	0.00002	1.68	0.00000
O55026	Ectonucleoside triphosphate diphosphohydrolase 2	Entpd2	0.60	0.00061	1.54	0.00162
A2AQP0	Myosin-7B	Myh7b	0.76	0.00048	1.26	0.00088
Q62479	X/Y protein (Fragment)	Cr1l	0.59	0.00001	1.51	0.00006
P61358	60S ribosomal protein L27	Rpl27	0.48	0.00000	1.79	0.00001
Q01768	Nucleoside diphosphate kinase B	Nme2	0.68	0.00870	1.41	0.00173
Q9CZP6	KH type-2 domain-containing protein	Rps3	0.58	0.00000	1.37	0.00000
Q05CG9	Psmd1 protein (Fragment)	Psmd1	0.43	0.00000	1.38	0.00005
Q64310	Surfeit locus protein 4	Surf4	0.54	0.00001	1.52	0.00013
Q3TK56	Actin-related protein 2/3 complex subunit 5 (Fragment)	Arpc5	0.54	0.00001	1.32	0.00064
Q5SUC3	Uncharacterized protein	Canx	0.49	0.00001	1.65	0.00002
P08249	Malate dehydrogenase, mitochondrial	Mdh2	0.51	0.00059	1.37	0.00018
Q99KI3	ER membrane protein complex subunit 3	Emc3	0.59	0.00001	1.46	0.00011
Q9D517	1-acyl-sn-glycerol-3-phosphate acyltransferase gamma	Agpat3	0.40	0.00077	1.77	0.00012
Q3TFP8	Cytochrome b5 heme-binding domain-containing protein	Pgrmc1	0.47	0.00105	1.43	0.00000
D3Z041	Long-chain-fatty-acid--CoA ligase 1	Acsl1	0.49	0.00003	1.84	0.00000
A0A087WS16	Collagen, type VI, alpha 3	Col6a3	0.56	0.00001	1.67	0.00001
Q8BFZ1	Trans-2,3-enoyl-CoA reductase-like	Tecrl	0.43	0.00189	1.41	0.00001
Q5H8C4	Vacuolar protein sorting-associated protein 13A	Vps13a	0.62	0.01064	1.35	0.03226

Q9CXG2	Uncharacterized protein	Rab22a	0.47	0.00013	1.37	0.00545
P16546-2	Isoform 2 of Spectrin alpha chain, non-erythrocytic 1	Sptan1	0.55	0.00077	1.59	0.00311
P27773	Protein disulfide-isomerase A3	Pdia3	0.63	0.00013	1.32	0.00144
A0A0U1RPS0	AP-2 complex subunit sigma	Ap2s1	0.47	0.00000	1.87	0.00000
P22599	Alpha-1-antitrypsin 1-2	Serpina1b	0.49	0.00000	1.56	0.00002
Q9DBS1	Transmembrane protein 43	Tmem43	0.52	0.00000	1.61	0.00099
Q9QZV4	Histidine-rich Ca2+ binding protein	Hrc	0.44	0.00001	1.72	0.00004
Q8K126	Peptide chain release factor 1, mitochondrial	Mtrf1	0.44	0.00000	1.45	0.00008
Q3U3Q7	Angiotensin-converting enzyme	Ace	0.48	0.00409	1.76	0.00007
Q8BJ03	Cytochrome c oxidase assembly protein C	Cox15	0.35	0.00000	1.70	0.00008
Q3UGY4	Spectrin beta chain	Sptb	0.51	0.00000	1.48	0.00007
Q8K2B3	Succinate dehydrogenase [ubiquinone] flavoprotein subunit, mitochondrial	Sdha	0.47	0.00001	1.76	0.00254
A0A2R8VI30	Leukocyte surface antigen CD47	Cd47	0.49	0.00000	1.39	0.00000
Q9DCT8	Cysteine-rich protein 2	Crip2	0.43	0.00069	1.43	0.00000
Q9R0L6	Pericentriolar material 1 protein	Pcm1	2.95	0.00124	0.76	0.00997
Q922Q7	Cathepsin H	Ctsh	0.56	0.00159	1.52	0.00329
O54724	Caveolae-associated protein 1	Cavin1	0.42	0.00001	1.47	0.00084
Q8R3G1	Nuclear inhibitor of protein phosphatase 1	Ppp1r8	1.41	0.00024	0.54	0.00002
O88952	Protein lin-7 homolog C	Lin7c	0.45	0.00000	1.69	0.00638
P26041	Moesin	Msn	0.57	0.00001	1.41	0.00000
A0A0R4J1G9	Metalloreductase STEAP3	Steap3	0.50	0.00000	1.49	0.00015
A0A140T8T4	Ribosomal protein L9, pseudogene 6	Rpl9-ps6	0.50	0.00000	0.30	0.00000
Q5CZX6	Acbd5 protein (Fragment)	Acbd5	0.45	0.00000	1.59	0.00001
Q9D8B7	Junctional adhesion molecule C	Jam3	0.44	0.00000	1.73	0.00000
Q9DCN2	NADH-cytochrome b5 reductase 3	Cyb5r3	0.72	0.00004	1.31	0.00002
Q9CZW5	Mitochondrial import receptor subunit T	Tomm70	1.35	0.00008	0.73	0.00015
Q8BMS1	Trifunctional enzyme subunit alpha, mitochondrial	Hadha	1.81	0.00004	0.65	0.00001
Q9JKR6	Hypoxia up-regulated protein 1	Hyou1	0.53	0.00000	1.34	0.00025
A6ZI44	Fructose-bisphosphate aldolase	Aldoa	0.70	0.00048	1.46	0.00064
Q61743	ATP-sensitive inward rectifier potassium channel 11	Kcnj11	0.42	0.00001	1.30	0.00165

[a] The FC (Fold Change) was calculated from the average abundances (Normalized peak area) of DOX group vs. control group and SGI-treated group vs. DOX group. [b] The *p*-value was calculated from Student's two-tailed t-test.

Supplementary Figures

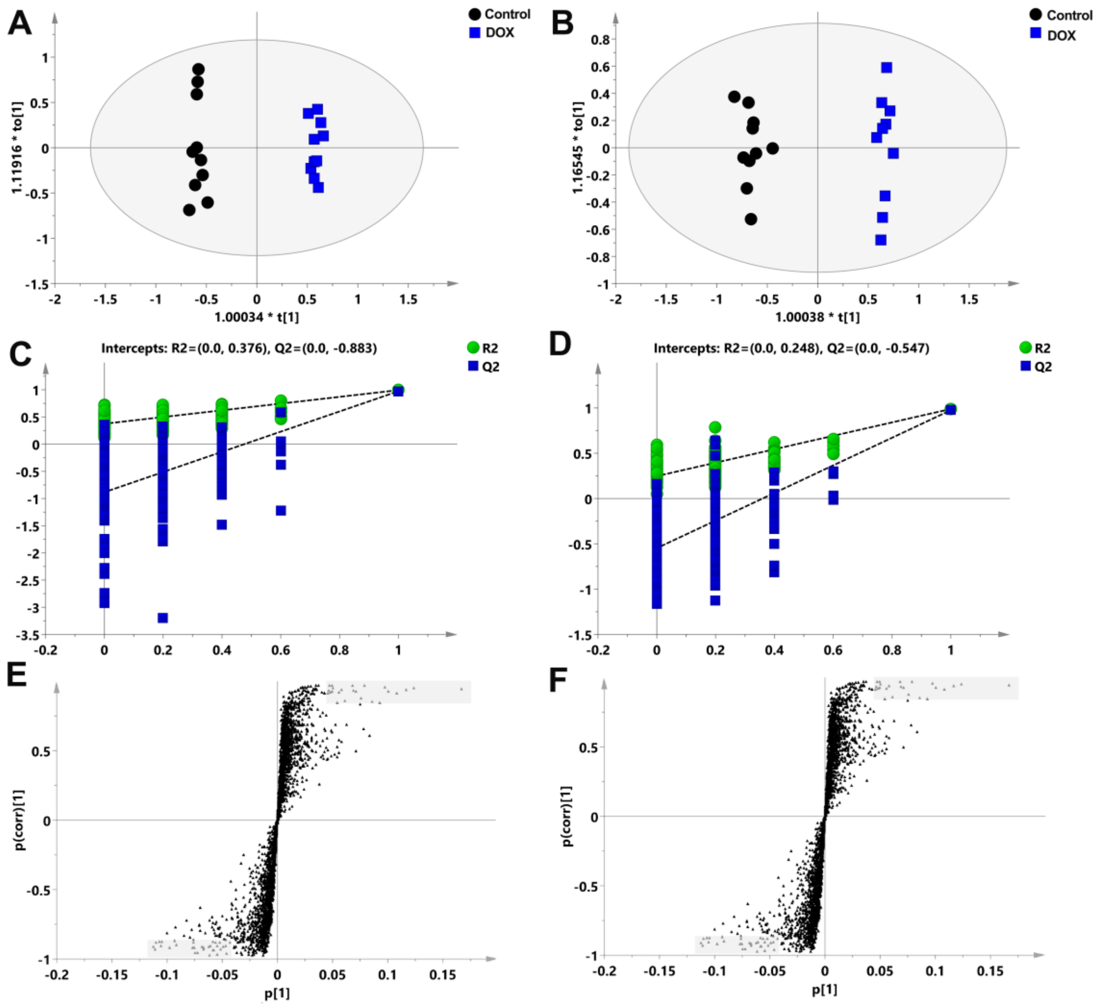

Figure S1. The OPLS-DA score plots from UHPLC-Q-TOFMS positive ion data (A) and negative ion data (B) of control and DOX groups. The quality was validated by 200 permutation tests for positive ion data(C) and negative ion data(D). S-plot of the OPLS-DA model of control and DOX groups from positive ion data (E) and negative ion data (F).

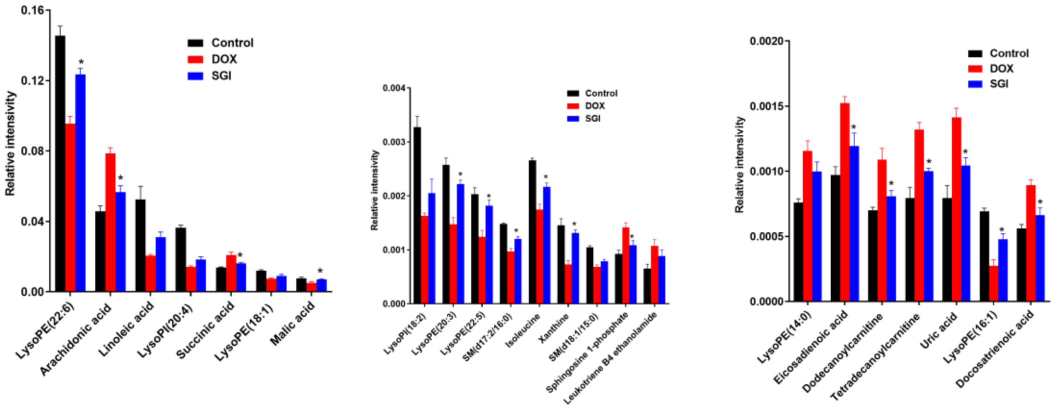

Figure S2. Changes in the relative concentrations of target metabolites identified. ANOVA was used to determine the significance of the change in relative concentrations for each metabolite. 16 metabolites were significantly reversed by SGI treatment. Bars represent the mean relative metabolite concentration and standard deviations. * $p < 0.05$ versus DOX group.

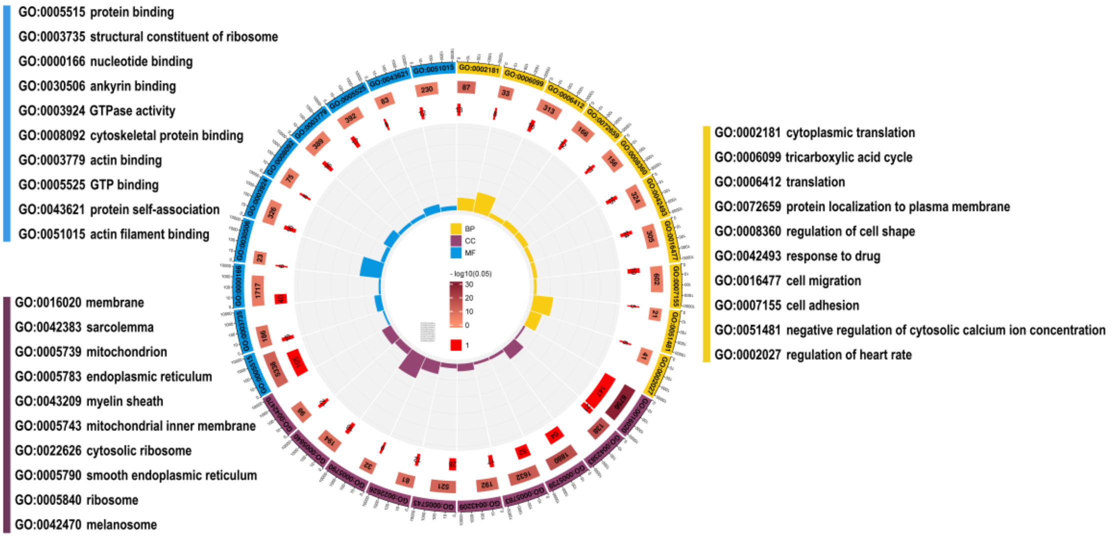

Figure S3. Circle diagram of Gene Ontology (GO) enrichment analysis of DEPs. Yellow, blue, and brown represent biological process (BP), molecular function (MF), and molecular function (CC), respectively. The first lap indicates the top 10 GO terms of each category. The second lap indicates the number of the genes in the genome background and P-values for gene enrichment for the specified GO terms. The third lap indicates bar graph of enriched genes. The fourth lap indicates the enrichment factor for each GO term.

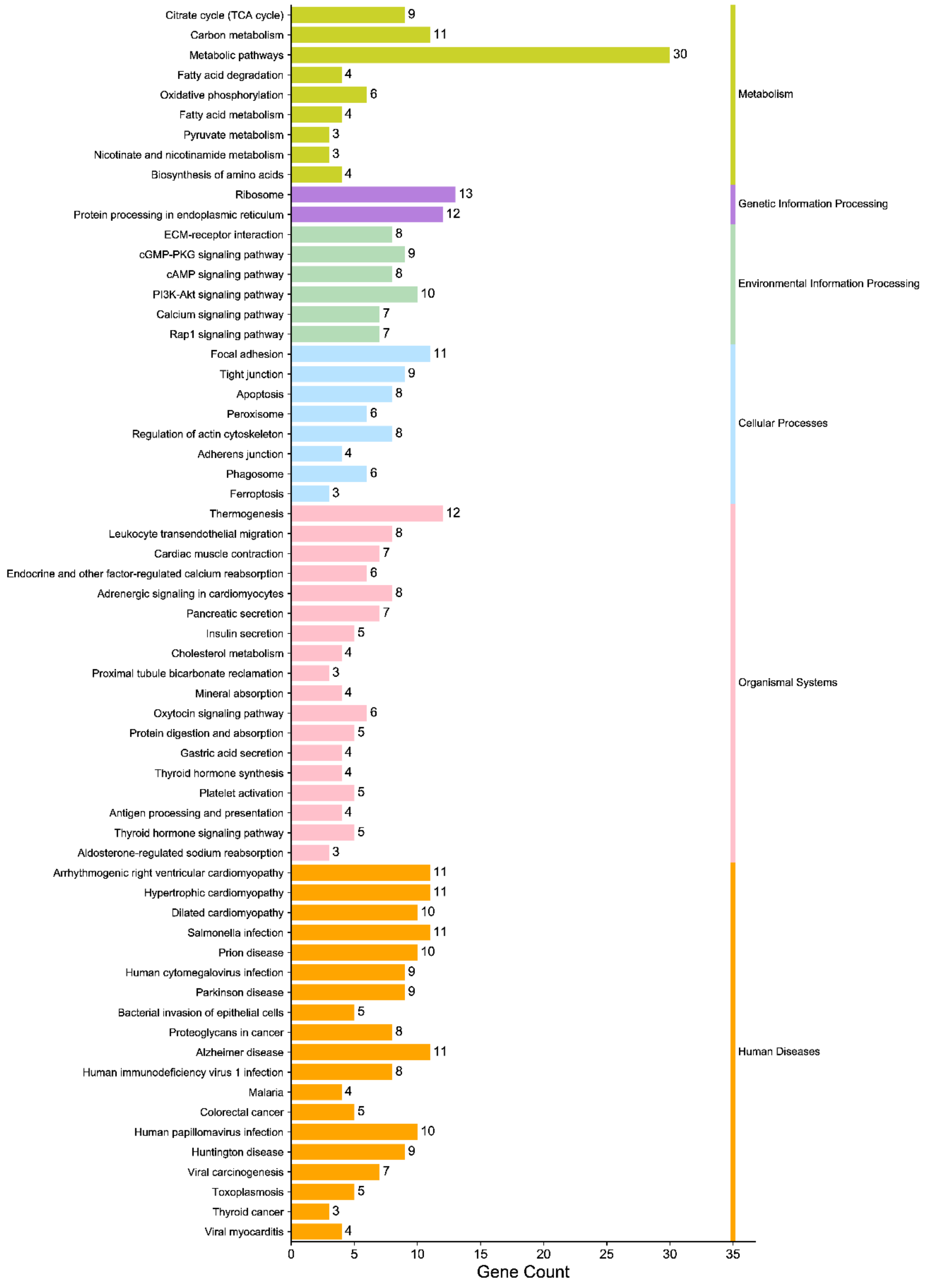

Figure S4. The DEPs are associated with diseases and signal transduction pathways by KEGG enrichment. The number of proteins associated with each category is listed.

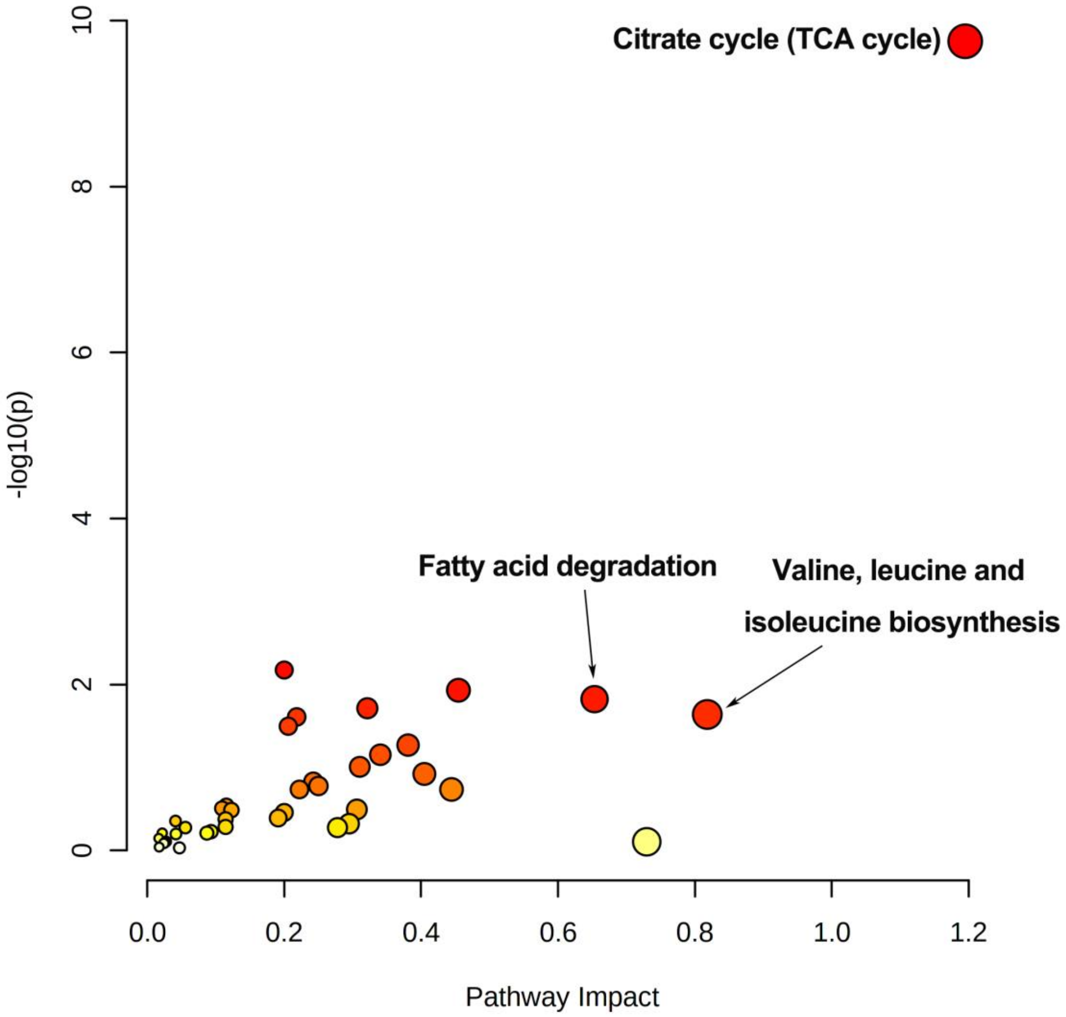

Figure S5 Integrated metabolic pathway enrichment analysis of differential metabolites and proteins by MetaboAnalyst 5.0.
